# Dynamic Evolution of the *Cthrc1* Genes, a Newly Defined Collagen-Like Family

**DOI:** 10.1093/gbe/evaa020

**Published:** 2020-02-08

**Authors:** Lucas Leclère, Tal S Nir, Michael Bazarsky, Merav Braitbard, Dina Schneidman-Duhovny, Uri Gat

**Affiliations:** e1 Laboratoire de Biologie du Développement de Villefranche-sur-Mer (LBDV), Sorbonne Université, CNRS, Villefranche-sur-Mer, France; e2 Department of Cell and Developmental Biology, Silberman Life Sciences Institute, The Hebrew University of Jerusalem, Israel; e3 Department of Biochemistry, Silberman Life Sciences Institute, The Hebrew University of Jerusalem, Israel; e4 School of Computer Science and Engineering, The Hebrew University of Jerusalem, Israel

**Keywords:** Cthrc1, collagen, *Cnidaria*, *Nematostella*, *Clytia*, phylogeny, gene loss, protein structure modeling, C1q domain

## Abstract

Collagen triple helix repeat containing protein 1 (Cthrc1) is a secreted glycoprotein reported to regulate collagen deposition and to be linked to the Transforming growth factor *β*/Bone morphogenetic protein and the Wnt/planar cell polarity pathways. It was first identified as being induced upon injury to rat arteries and was found to be highly expressed in multiple human cancer types. Here, we explore the phylogenetic and evolutionary trends of this metazoan gene family, previously studied only in vertebrates. We identify *Cthrc1* orthologs in two distant cnidarian species, the sea anemone *Nematostella vectensis* and the hydrozoan *Clytia hemisphaerica*, both of which harbor multiple copies of this gene. We find that *Cthrc1* clade-specific diversification occurred multiple times in cnidarians as well as in most metazoan clades where we detected this gene. Many other groups, such as arthropods and nematodes, have entirely lost this gene family. Most vertebrates display a single highly conserved gene, and we show that the sequence evolutionary rate of *Cthrc1* drastically decreased within the gnathostome lineage. Interestingly, this reduction coincided with the origin of its conserved upstream neighboring gene, *Frizzled 6* (*FZD6*), which in mice has been shown to functionally interact with Cthrc1. Structural modeling methods further reveal that the yet uncharacterized C-terminal domain of Cthrc1 is similar in structure to the globular C1q superfamily domain, also found in the C-termini of collagens VIII and X. Thus, our studies show that the *Cthrc1* genes are a collagen-like family with a variable short collagen triple helix domain and a highly conserved C-terminal domain structure resembling the C1q family.

## Introduction

**Fig. 1.**
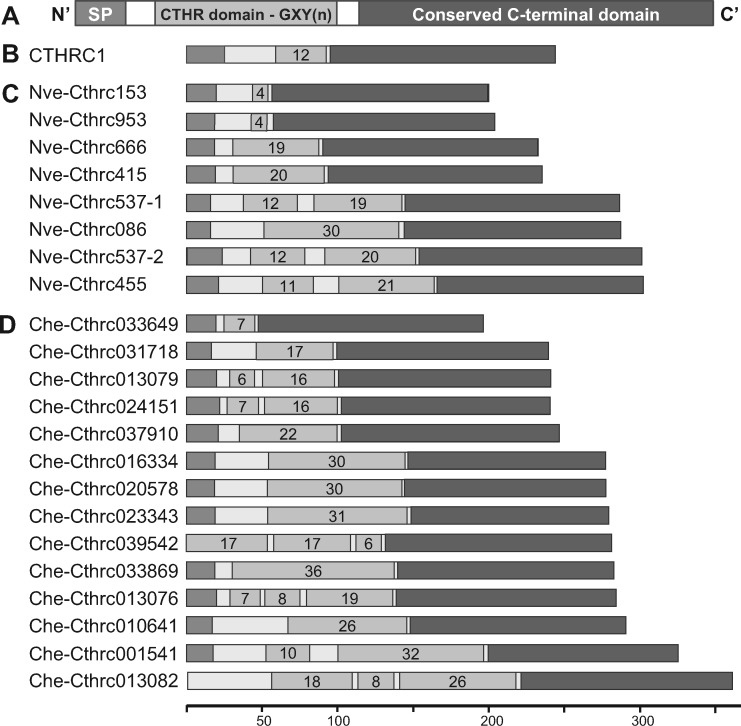
—Structure of the *Nematostella* and *Clytia* Cthrc1 protein families. (*A*) A general scheme of a Cthrc1 protein which is composed of a signal peptide (SP), a collagen triple helix repeat domain (CTHR or GXY(*n*)) and a conserved C-terminal domain. Protein structure of the (*B*) human CTHRC1 (with GXY *n* = 12), (*C*) 8 *Nematostella vectensis Cthrc1*, and (*D*) 14 *Clytia hemisphaerica Cthrc1* gene products with the number of GXY repeats depicted on the CTHR domains. The N-terminal part is missing for two *Clytia* predicted Cthrc1 proteins (Che-Cthrc039542 and Che-Cthrc013082). The bottom scale bar indicates the length in amino acids for the sequences shown in (*B*)–(*D*). See supplementary table S1 and figs. S1 and S2, Supplementary Material online, for more details.

The *collagen triple helix repeat containing 1* (*Cthrc1*) gene was first reported by the Lindner laboratory in a screen for genes differentially expressed upon major damage to arteries in rats (Pyagay et al. 2005). C*thrc1* was highly induced in injured arteries, with expression subsiding upon healing (Pyagay et al. 2005; LeClair et al. 2007). The protein was predicted to have an N-terminal hydrophobic signal peptide, followed by a typical collagen triple helix repeat (CTHR) domain, consisting of 12 GXY repeats in mammals, and a conserved C-terminal domain without known homology to other proteins (Pyagay et al. 2005) (fig. 1). Biochemical analyses showed that the protein is N-glycosylated, forms trimers by virtue of its CTHR regions shown to be susceptible to collagenase digestion, and is likely secreted (Pyagay et al. 2005).

Cthrc1 was demonstrated to play a role in tissue remodeling and morphogenesis by promoting cell migration and by reducing the deposition of the collagen matrix (Pyagay et al. 2005), likely through TGFβ (Transforming growth factor β) signaling regulation. The gene was found to be expressed, in mouse, at sites of interstitial collagen deposition, which are known to be hotspots of high TGFβ activity; such expression was reported for both embryonic tissues, including the notochord, and for several adult organs such as skeleton, heart, and kidney (Durmus et al. 2006). *Cthrc1* was induced by TGFβ and BMP4 (Bone morphogenetic protein 4) factors in cell assays (Pyagay et al. 2005), and a putative Smad binding site was identified in the gene’s presumed promoter region (Tang et al. 2006). Later reports demonstrated that Cthrc1 can in turn inhibit TGFβ signaling both in vitro and in vivo by inducing phospho-Smad3 degradation (LeClair and Lindner 2007; LeClair et al. 2007). In zebrafish, Cthrc1 was recently shown to play an essential role in epiboly and convergent-extension cell movements during gastrulation by regulating cell migration and integrin-mediated cell adhesion (Cheng et al. 2019).

*CTHRC1* was reported to be aberrantly expressed in multiple human cancers and to be functionally associated with cancer cell migration, tumor invasiveness, and metastasis (reviewed by Tang et al. [2006] and Jiang et al. [2016]). High expression of *CTHRC1* was detected in many human solid tumors such as of the ovary, liver, and pancreas (Allinen et al. 2004; West et al. 2005). *CTHRC1* expression could be correlated with melanoma cell lines and tumors migration, invasiveness, and metastasis abilities, whereas *CTHRC1* knockdown in melanoma cell lines leads to a decrease in cell migration (Tang et al. 2006). However, monoclonal antibodies could not detect CTHRC1 protein in multiple cancerous cell types, suggesting that in those cases, the cells surrounding the tumor and not the cancer cells are expressing the protein(Duarte et al. 2014).

The Wnt planar cell polarity (PCP) pathway is a noncanonical Wnt signaling pathway, involved in several morphogenetic processes during development, affecting in particular concerted cell movements and cell polarity within tissues (Yang and Mlodzik 2015). Cthrc1 selectively activates the Wnt/PCP pathway by stabilizing Wnt-FZD/Ror2 ligand–receptor interaction, as first demonstrated by the Sasaki Laboratory (Kelley 2008; Yamamoto et al. 2008). In this work, *Cthrc1* expression was identified in the inner ear of mice and the knockout of this gene was found to give rise to PCP phenotypes (such as the misorientation of the sensory hair cells within the cochlea) when crossed with a *Vangl2* mutant line (Yamamoto et al. 2008). It was also demonstrated that Cthrc1 binds Wnt cofactors, frizzled receptors, and the Wnt/PCP-specific Ror2 coreceptor and that it enhances Wnt/PCP pathway activation and inhibits the canonical Wnt/β-catenin pathway. Despite the fact that activation of Wnt/PCP by Cthrc1 has recently been questioned (Jin et al. 2017), several reports have demonstrated this interaction in colorectal cancer cells (Yang, You, et al. 2015), gastrointestinal stromal tumors (Ma et al. 2014), and in mouse hair follicles as well, where Cthrc1 was shown to bind Frizzled 6 (FZD6) and to enhance Wnt/PCP-induced Rho activation (Dong et al. 2018). The expression of Cthrc1 was also found to be induced by the FZD6 but not FZD3-mediated Wnt/PCP activation (Dong et al. 2018).

The *Cthrc1* gene was first reported only in vertebrates and in the ascidian *Ciona intestinalis* (Pyagay et al. 2005). It was then mentioned to be present in the sponge *Oscarella carmela* (Nichols et al. 2006) as well as to be enriched in the colony branch tips of staghorn corals (Hemond et al. 2014). No systematic phylogenetic study of these genes has been reported to date. In this work, we characterized the phylogenetic distribution of these genes and compared their sequences and gene structure across Metazoa. Using structure modeling methods, we further reveal the similarity of the previously undefined Cthrc1 C-terminal domain to the C1q (complement component 1q) domain family, and notably to the C1q domain-like “noncollagen” (NC) domains of the network forming collagens VIII and X. We demonstrate the dynamic evolution of this collagen-like gene family with multiple lineage-specific diversifications and losses and shed light on its structural identity.

## Materials and Methods

### Sequence Identification and Phylogenetic and Synteny Analyses

The *Nematostella vectensis Cthrc1* genes were searched by BLAST analysis (TBlastN) on the JGI genome database (Putnam et al. 2007) using the human isoform 1 CTHRC1 sequence (NP_612464.1) and the Nve-Cthrc666 (NEMVEDRAFT_v1g216666) protein sequences to find candidate sequences for other family members. The genomic sequences and their predicted mRNA transcripts from NCBI thus obtained were compared with full-length transcript sequences located in the available transcriptome databases which included the *N.* *vectensis* transcriptome and gene models v2.0 (nveGenes: https://doi.org/10.6084/m9.figshare.807694.v1; last accessed February 14, 2020), Stellabase (Sullivan et al. 2006), the transcriptome shotgun assembly of the Martindale laboratory (Babonis et al. 2016), and the NvERTx embryonic and regenerative transcriptome exploration tool (Warner et al. 2018). Eight full-length *Nve-Cthrc1* genes were assembled by extracting for each at least one full-length transcript from the above databases and verifying its sequence by using BLAST (BlastN) on the *Nematostella* JGI genome database; the other JGI genomic entries which encompassed identical albeit partial sequences of these genes were discarded. The *Clytia hemisphaerica Cthrc1* genes were retrieved by BLAST (TBlastN) from the current genome/transcriptome assemblies (Leclère et al. 2019) using the human Cthrc1 and the predicted *Nematostella* Cthrc1 sequences as queries. *Clytia* Cthrc1 sequence containing only partial CTHR or C-terminal domains (five predicted proteins) were excluded.

Cthrc1 sequences from other species were searched by BlastP and TBlastN through the NCBI BLAST interface (v.2.10.0+, *e*-value < 0.01) on available proteins, genomes, and transcriptomes (nonredundant nr v.2019/03/22, nt v.2019/10/03) as well as on dedicated databases (see supplementary tables S2 and S3, Supplementary Material online, for details) using at first the C-terminal domain of human Cthrc1 and Nve-Cthrc666 as query sequences, and for non-Metazoa also using the Cthrc1 C-terminal domain sequences found in a dinoflagellate (OLQ02974) and in a bacteria metagenome (RKZ59233). A PANTHER entry (PTHR11903: SF18) corresponding to the C-terminal domain of Cthrc1 was found in the INTERPRO database, using the same four query sequences, confirming the dinoflagellate and bacteria sequences identified in our blast searches (see supplementary table S2, Supplementary Material online). FZD6/FZD3 and Slc25a32 homologs were identified by BlastP through the NCBI BLAST interface on a selection of metazoans using the mouse FZD6 and Slc25a32 as query sequence respectively (see Supplementary Material online). Other Frizzled proteins from Human, *Saccoglossus kowaleski*, and *Branchiostoma belcheri* were included as outgroup in the FZD3/6 sequence alignment.

Sequences were aligned using the MAFFT v7.271 L-INS-I algorithm (Katoh et al. 2002). Cthrc1 sequences with incomplete C-terminal domain were excluded. Positions with more than 50% gaps were manually excluded to produce the final alignments. Only the Cthrc1 C-terminal domain was used for the phylogenetic analyses of cnidarian and metazoan proteins, because the CTHR domain, composed of a highly variable number of GXY repeats, could not be aligned accurately (see supplementary alignment files, Supplementary Material online). C-terminal and CTHR domains were included in the phylogenetic analyses of vertebrate Cthrc1 as they could be unambiguously aligned. The CRD and 7tm domains of the Frizzled proteins were split after alignment and each domain was used separately for phylogenetic analyses. Untrimmed and trimmed alignments used for phylogenetic analyses are available as supplementary files, Supplementary Material online.

Maximum likelihood (ML) analyses were performed using RaxML v8.2.9 (Stamatakis 2006) on a Linux server using the parallel version raxmlHPC-PTHREADS-AVX (random seed for all analyses: “-p 2346”). The best fitting model was first evaluated for each alignment using the PROTGAMMAAUTO command (metazoan Cthrc1 alignments: PROTGAMMAWAG; Cthrc1-C-terminal domain eukaryote/bacteria alignment: PROTGAMMALG; Frizzled and Slc25a32 alignments: PROTGAMMAJTT). Bootstrap support values were calculated from 500 replicates using the best fitting model (commands: “-# 500 -x 12345”). Bootstrap values were then drawn on the best ML tree (command: “-f b”). Bio-NJ analyses (kimura distance matrix) including bootstrap analyses (500 replicates) were performed using SeaView v4.7 (Gouy et al. 2010).

The genes located immediately upstream and downstream of Cthrc1 were identified in human, *Mus musculus*, *Gallus gallus*, *Xenopus laevis*, *Cyprinus carpio*, *Danio rerio*, *Salmo salar*, *Takifugu rupestris*, *Callorhinchus milii*, *Petromyzon marinus*, and *Ci.* *intestinalis* using the Ensembl, NCBI, and ANISEED databases (Brozovic et al. 2018) (see supplementary table S3, Supplementary Material online). For each analyzed species, the genomic location of the previously identified *FZD6* and *Slc25a32* genes (see above) was determined using the corresponding genome databases.

### Ab Initio Modeling

Methods of protein domain structure prediction, termed ab initio structural modeling, can be employed in the absence of homologs with a solved 3D structure, such as for Cthrc1 here studied. The sequences of the C-terminal domain of Cthrc1 from *N.* *vectensis* and from human were used as the input to the Rosetta ab initio Relax protocol (Simons et al. 1997) with the following parameters: increase cycles 20, rg_reweight 0.5, rsd_wt_helix 0.5, and rsd_wt_loop 0.5. Approximately 60,000 models were generated and ranked using the Rosetta scoring function. Simultaneously, we submitted the sequences to the I-TASSER server with the default parameters and received five models. The top-scoring models from both programs were structurally aligned to the structures of the C-terminal noncollagenous (NC1) domains of mouse collagen Alpha1 (VIII) (protein data bank [PDB]: 1o91), the human collagen X NC1 (PDB: 1gr3), and the BclA protein (PDB: 1wck), to the globular C-terminus of protein C1q (PDB: 1pk6), the COLFI domain of fibrillar procollagen type III (PDB: 4ae2), and to the C-terminus of collagen type IV (PDB: 5nay). The models with the largest number of aligned Cα atoms were selected. The best models (best scoring model and largest alignment model from Rosetta and the models from I-TASSER, for every sequence) were ranked using statistically optimized atomic potentials (SOAP) (Dong et al. 2013). We chose the models that have low SOAP scores (<−80,000) and over 50% structural similarity to the collagen-like or C1q structures.

## Results

The *Cthrc1* gene was discovered in mammals and functionally studied only in vertebrates up until this study. We found *Cthrc1-*related genes in two distantly related cnidarian species, *N.* *vectensis* and *C.* *hemisphaerica*. We first studied the phylogenetic distribution of this gene family across cnidarians, before expanding the analysis to all metazoans, uncovering a large number of clade-specific gene duplications, complete losses, as well as strong sequence and synteny conservation in vertebrates. We further characterized these collagen-like proteins through in silico structural modeling.

### The Genomes of the Cnidarians *Nematostella* and *Clytia* Contain Multiple *Cthrc1* Genes

In the course of a whole-body regeneration transcriptional screen in the sea anemone *N.* *vectensis* (Schaffer et al. 2016), we discovered a gene family with high sequence identity to mammalian *Cthrc1*. Analyses of the available *Nematostella* genome (Putnam et al. 2007) and transcriptomes (see Materials and Methods) identified eight bona fide members of the *Cthrc1* gene family (*Nve-Cthrc1*’*s*). We then searched the genome of the recently sequenced hydrozoan model *C.* *hemisphaerica* (Leclère et al. 2019) and found 14 additional members (*Che-Cthrc1*’*s*). As in vertebrates, the predicted proteins comprise a signal peptide, a CTHR domain, and a conserved C-terminal domain (fig. 1 and supplementary figs. S1 and S2 and table S1, Supplementary Material online). The *Clytia* and *Nematostella* paralogs are nevertheless quite diverse in terms of sequence and differ greatly in the composition and length of the CTHR domain, ranging from 4 repeats in two of the Nve-Cthrc1 to 54 repeats in one of the Che-Cthrc1 (fig. 1 and supplementary table S1, Supplementary Material online). In a few paralogs, the CTHR domain is interrupted by short non-GXY sequences. Several introns were identified, with most *Che-Cthrc1*’s and *Nve-Cthrc1*’s harboring at least two. Interestingly, the position of the intron at the 5′ region of the C-terminal coding domain is conserved between *Nematostella*, *Clytia*, and all the metazoans for which genomic data were checked, with the exception of *Ci.* *intestinalis* (supplementary fig. S3, Supplementary Material online).

**Fig. 2.**
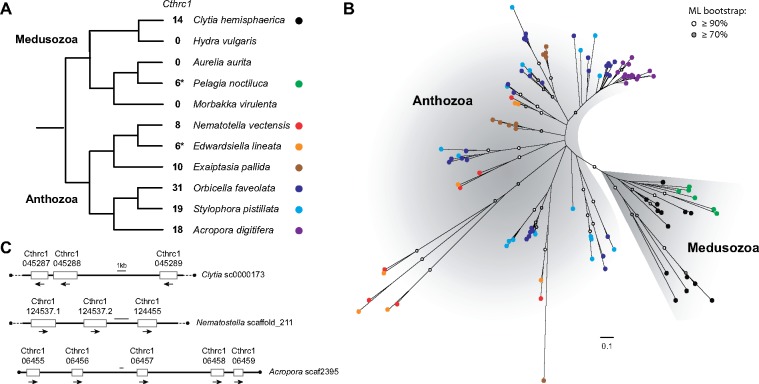
—Phylogeny of the cnidarian *Cthrc1* genes. (*A*) Phylogenetic relationships of the sampled cnidarian species, with the number of *Cthrc1* genes per species. *Number of Cthrc1 determined from transcriptomic data only. (*B*) Unrooted ML phylogeny (model: WAG + G) of the sampled cnidarian Cthrc1 using the C-terminal domain. ML bootstrap support values (500 replicates) are shown as circles on branches: white circles ≥90% and gray circles ≥70%. Scale bar: estimated number of substitutions per site. The color of the circles at the branch tips indicates the species as depicted in (*A*). See supplementary table S3 and fig. S4, Supplementary Material online, for more details. (*C*) Examples of genomic scaffolds containing several tandem duplicated *Cthrc1* genes as found in *Clytia hemisphaerica*, *Nematostella vectensis*, and *Acropora digitifera*.

Only the highly conserved C-terminal domain was used as query in the BLAST searches aimed at further exploring the cnidarian *Cthrc1* repertoire, due to the large variation in the size of the CTHR domain, which confounded the results. Phylogenetic analyses of the *Cthrc1* C-terminal domain from *Nematostella*, *Clytia*, and a selection of cnidarian species, strongly suggest that *Cthrc1* diversification occurred mostly independently in corals, sea anemones, hydrozoans, and scyphozoans. Only a few orthology relationships could indeed be convincingly identified between these four groups. The weak phylogenetic support of many branches, perhaps due to the shortness of the *Cthrc1* C-terminal domain (130–150 amino acids), hinders a precise reconstruction of the evolutionary history of cnidarian *Cthrc1* genes. We could nevertheless conclude with confidence that the *Cthrc1* paralogs found in *Clytia* and *Nematostella* originated from distinct diversification events (99% bootstrap support—BP—fig. 2*A* and *B*) and that cnidarian *Cthrc1* diversification was the result of both old and more recent events. Several phylogenetically closely related *Cthrc1* paralogs in *Clytia, Nematostella*, and in the coral *Acropora digitifera* (*Adi*) (100% BP for each) were found in close proximity on the same genomic scaffold and likely resulted from tandem duplications (fig. 2*C*). Comparison of *Cthrc1* sequences between *N.* *vectensis* and its close relative *Edwardsiella lineata* (*Edw*) allowed defining orthology groups (e.g., *Nve_Cthrc537-2* and *Edw_T1_41245–*100% BP) (fig. 2*B* and supplementary fig. S4, Supplementary Material online), indicating that most duplication events leading to the formation of the eight *Nve-Cthrc1* were older than their last common ancestor, estimated at 184–213 Ma (Dnyansagar et al. 2018). Within sea anemones, we could only detect one weakly supported orthology relationship between *Nematostella* and *Exaiptasia pallida Cthrc1* genes (Baumgarten et al. 2015) (*Nve-Cthrc086* and *Epa_XP_020897757–*48% BP) (fig. 2*B* and supplementary fig. S4, Supplementary Material online). We also identified an orthology group (albeit weakly supported) shared between sea anemones and corals *(Nve-Cthrc086* and *Spi_XP_022784555–*66% BP) (fig. 2*B* and supplementary fig. S4, Supplementary Material online), which would be thus older than the estimated divergence between these groups, 540–600 Ma (Hedges et al. 2015, but see Dohrmann and Worheide [2017] for older time estimates).

### Frequent Diversifications and Losses of *Cthrc1* Genes in Metazoa

**Fig. 3.**
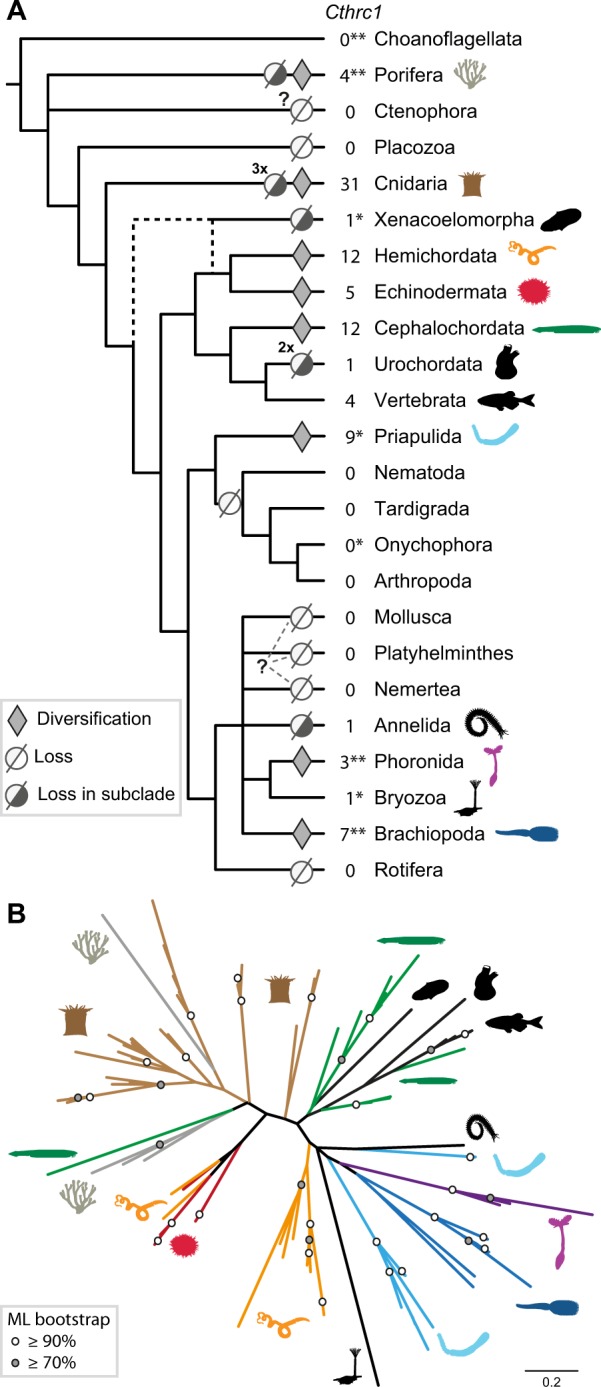
—Diversifications and losses of Cthrc1 in Metazoa. (*A*) Evolution of Cthrc1 reconstructed onto the accepted metazoan phylogenetic tree. The numbers provided indicate the highest number of *Cthrc1* genes found in the sampled species of the corresponding clade (details in supplementary table S3, Supplementary Material online). Cthrc1 was lost at least twice (2×) in urochordates and at least three times (3×) in medusozoans. (?) Unsettled phylogenetic position of Ctenophora, Nemertea, Platyhelminthes, and Mollusca prevented inferring whether Cthrc1 was lost in the common ancestor of these clades. *Number of Cthrc1’s determined from transcriptomic data only. **Sequences containing a Cthrc1 C-terminal domain without the CTHR domain. See supplementary table S3, Supplementary Material online, for more details. (*B*) Unrooted ML phylogeny (model: WAG + G) of the Cthrc1 C-terminal domain from a selection of metazoans. For each group list in (*A*), all identified Cthrc1 sequences of the most Cthrc1-rich species were included. ML bootstrap support values (500 replicates) are shown as circles on branches: white circles ≥90% and gray circles ≥70%. Colors and silhouettes in (*B*) correspond to those shown in (*A*). Scale bar: estimated number of substitutions per site. The animal silhouettes are from Phylopic. See supplementary tables S2 and S3 and fig. S6, Supplementary Material online, for more details.

The further exploration of the *Cthrc1* repertoire across eukaryotes, by means of broad genome and transcriptome analyses, revealed at first that *Cthrc1* is a metazoan-specific gene (sensu CTHR combined to Cthrc1 C-terminal domain). Sequences resembling Cthrc1 C-terminal domain—but lacking the CTHR domain—could nevertheless be identified in several choanoflagellate transcriptomes (Richter et al. 2018) (fig. 3*A* and supplementary table S2, Supplementary Material online) as well as in *Symbiodinium* dinoflagellates genomes and transcriptomes (supplementary fig. S5, Supplementary Material online). Cthrc1 C-terminal domain could not be found in ichthyosporeans (Torruella et al. 2015), in the filasterean *Capsaspora owczarzaki* (Suga et al. 2013), or in the genomes of the choanoflagellates *Monosiga brevicollis* (King et al. 2008) and *Salpingoeca rosetta* (Fairclough et al. 2013) where other supposedly “metazoan-specific” genes were later detected (Sebe-Pedros et al. 2017) (supplementary table S2, Supplementary Material online)

Interestingly, we could find Cthrc1 C-terminal domain in several bacteria metagenomes, including some predicted proteins which also contain a CTHR domain (supplementary fig. S5, Supplementary Material online). Phylogenetic analyses of the Cthrc1 C-terminal domain across eukaryotes and bacteria supported a grouping of dinoflagellate and bacteria sequences with choanoflagellate sequences (92% BP). Importantly, the bacterial sequences featuring both Cthrc1 C-terminal and CTHR domains were nested within bacteria (100% BP); their CTHR domain showed highest similarity with other collagen-like bacterial sequences (BlastP *e*-value: 2e-14), strongly arguing in favor of an independent combination of the CTHR and Cthrc1 C-terminal domains in Metazoa and Bacteria.

We then performed an extensive search across metazoans and identified multiple *Cthrc1* genes in several poriferan, cnidarian, and bilaterian clades but none in Ctenophora and Placozoa. The patchy distribution of the *Cthrc1* genes across Metazoa suggests multiple loss events (fig. 3*A* and supplementary table S3, Supplementary Material online). From the pattern of presence/absence, we could infer a minimum of 12 independent losses of *Cthrc1* genes in Metazoa (fig. 3*A*). *Cthrc1* was likely lost in Placozoa, in the medusozoan cnidarian *Hydra* (Chapman et al. 2010), in the jellyfish *Aurelia* (Gold et al. 2019) and *Morbakka virulenta* (Khalturin et al. 2019), and in the demosponge *Amphimedon queenslandica* and in the acoel *Hofstenia miamia* (Gehrke et al. 2019). Losses were especially prevalent among protostomes, where relatively few groups possess *Cthrc1* genes, with at least one loss within Ecdysozoa (absent in Panarthropoda, Tardigrada, and Nematoda) as well as losses in annelids and in Rotifera. In addition, we could not identify *Cthrc1* in any platyhelminth, molluskan, and nemertean available genomes. In urochordates, *Cthrc1* was lost in appendicularians as well as in the colonial species *Botryllus schlosseri* and *Botrylloides leachii*, whereas it was found in the transcriptome of closely related ascidian species (Alie et al. 2018).

Most nonvertebrate species harbor several *Cthrc1* paralogs. The CTHR and the C-terminal Cthrc1 domains have evolved quite rapidly in most of these groups, with high variation of the number and sequences of the collagen repeats. The metazoan *Cthrc1* phylogeny is poorly resolved, irrespective of the reconstruction method used (Bio-NJ or ML, fig. 3*B*), and it is not possible to infer whether one or several paralogs were present in the common ancestor of Planulozoa, Bilateria, Protostomia, and Deuterostomia. We could identify, however, well supported clade-specific diversifications in Medusozoa and Anthozoa (99% BP, fig. 2*B*), and in priapulid, phoronid, echinoderm, brachiopod, hemichordate (100% BP for each, fig. 3*B* and supplementary fig. S6, Supplementary Material online), and cephalochordate species (91% BP). Phylogenetic analyses of the *Cthrc1* genes from several cephalochordates (supplementary fig. S7, Supplementary Material online) showed that paralogs are shared between *Branchiostoma* and *Asymmetron* species, indicating that the diversification occurred before the last common ancestor of this clade estimated at about 46 Ma (Igawa et al. 2017).

The phylogenetic distribution of *Cthrc1* genes is complex. We detected multiple losses in several major metazoan clades, which is in contrast to the numerous diversification events identified in the groups that have retained *Cthrc1* genes. Consistently, the cnidarian species possessing *Cthrc1* genes present a relatively high number of gene family members (up to 31 in corals, the highest number across metazoans). Only a minority of protostomes possess *Cthrc1* genes (up to 9 in *Priapulus*), whereas loss of this gene family was rare among deuterostomes (cephalochordates harboring up to 12 copies), with vertebrates displaying a unique phylogenetic pattern.

### Slower Evolution and Higher Structural Conservation of *Cthrc1* in Vertebrates

**Fig. 4.**
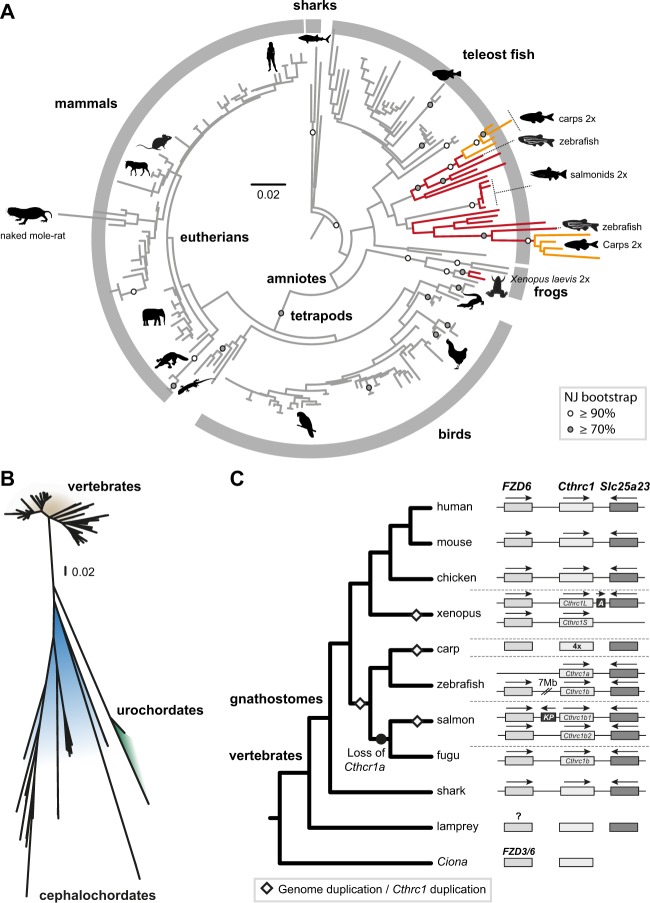
—Phylogeny of vertebrate Cthrc1. Unrooted NJ phylogeny (model: Kimura) of the Cthrc1 C-terminal domain including all available complete (*A*) gnathostome and (*B*) chordate sequences. See supplementary figs. S7–S10, Supplementary Material online, for more details. (*C*) Evolution of the *FZD6-Cthrc1-Slc25a32* synteny block across vertebrates. In zebrafish, *Cthrc1b* and *FZD6* are both found on chromosome 16, 7 Mb apart. In Salmon, keratin ultra-high-sulfur matrix protein-like gene (*KP*) is found between *Cthrc1a1* and *FZD6*. In *Xenopus laevis*, an *Acrosin-like* gene (*A*) is located between *Cthrc1L* and *Slc25a32*. The *FZD3/6* gene found in *Ciona* is orthologous to both *FZD6* and *FZD3*, whereas the phylogenetic position of the *FZD3/6*-like genes found in lamprey could not be assigned with confidence (see supplementary fig. S10, Supplementary Material online). Mouse, *Mus musculus*; Chicken, *Gallus gallus*; Xenopus, *Xenopus laevis*; Carp, *Cyprinus carpio*; zebrafish, *Danio rerio*; Salmon, *Salmo salar*; Fugu, *Takifugu rupestris*; Shark, *Callorhinchus milii*; Lamprey, *Petromyzon marinus*; and Ciona, *Ciona intestinalis*. In (*A*), the animal silhouettes are from Phylopic.

Although we inferred frequent duplication and gene loss events in many metazoan clades, this was not the case in vertebrates. We did not identify a single loss of the *Cthrc1* gene family in gnathostomes. In lamprey, only a partial *Cthrc1* sequence could be found. In most vertebrate species, only one *Cthrc1* gene could be identified, with up to four in teleost fishes. Vertebrate *Cthrc1* phylogeny reconstructed using the NJ algorithm is overall congruent with the accepted vertebrate species phylogeny, with monophyly recovered for the major vertebrate groups, such as teleosts, tetrapods, amniotes, frogs, mammals, and birds (fig. 4*A* and supplementary fig. S8, Supplementary Material online). ML analyses using the same alignments (see supplementary files, Supplementary Material online) are congruent with the NJ analyses but lead to obvious reconstruction artifacts, such as rooting of Cthrc1 vertebrate sequences within mammals, likely caused by the strong differences in sequence evolutionary rate between vertebrate and nonvertebrate Cthrc1.

From the analyses of Cthrc1 copy number in vertebrates, we concluded that from the two rounds of genome duplication at the base of the vertebrate tree (Dehal and Boore 2005), only one *Cthrc1* was retained in gnathostomes. Remarkably, all the vertebrate *Cthrc1* inferred gene duplications could be traced back to genome duplication events (fig. 4*A*). The two *Cthrc1* genes found in *Xenopus laevis* are related to the recent genome allotetraploidization event (Session et al. 2016). The two genes of several teleosts (Jaillon et al. 2004), including zebrafish and *Astyanax*, most likely resulted from the 3R teleost genome duplication. Eutelostei species, except Salmonids, have only a single *Cthrc1* gene indicating that one fish *Cthrc1* was likely lost in the last common ancestor of this group. The two genes found in Salmonidae and the four genes in Cyprinidae (carps) also likely resulted from more recent whole genome duplication events (Xu et al. 2014; Lien et al. 2016). In Carps, each of the two teleost *Cthrc1* paralogs was duplicated (fig. 4*A* and *C*).

Unlike for nonvertebrates, the Cthrc1 CTHR and the C-terminal domains were highly conserved during vertebrate evolution displaying no change in the number of GXY repeats (see supplementary files, Supplementary Material online). *Cthrc1* seems to have evolved markedly slower in gnathostomes than in other metazoan groups. Cthrc1 sequence differences between the whale shark and human, two species that diverged ∼465 Ma (Kumar et al. 2017), are less than those found between the two ascidian species *Ciona robusta* and *Ciona savignyi* that diverged ∼180 Ma (Berna and Alvarez-Valin 2014), or between paralogs of the same cephalochordate species, *Branchiostoma floridae*. Furthermore, the urochordate and cephalochordate Cthrc1, as well as the partial Cthrc1 C-terminal domain of the sea lamprey, show markedly longer branches than any gnathostome (fig. 4*B* and supplementary fig. S7, Supplementary Material online).

The rate of Cthrc1 sequence evolution in gnathostomes nevertheless increased in few lineages. Several *Cthrc1* genes of teleost fish, such as the zebrafish and carps paralogs (2 and 4 genes, respectively), show markedly longer branches (fig. 4*A*). This could be the result of neofunctionalization or subfunctionalization, known to cause higher evolutionary rates between paralogs (Pegueroles et al. 2013). The Cthrc1 of the naked mole-rat *Heterocephalus glaber*, also displays a considerably longer branch compared with its close relatives and other mammals (fig. 4*A*), suggesting a species-specific faster evolution.

Vertebrate *Cthrc1* not only is highly conserved at the sequence level but also belongs to a cluster of genes with shared synteny, which in most vertebrates consists of Cthrc1 being situated between *Frizzled6* (*FZD6*) and *Slc25a23* (fig. 4*C*). We could find FZD6 upstream of *Cthrc1* in the genome of almost all gnathostomes except in a few teleost fish, such as carps (different genomic scaffolds) and zebrafish (7 Mb apart). The mitochondrial folate carrier gene *Slc25a32* was found downstream of *Cthrc1* in all gnathostome genomes except in carps (fig. 4*C*). The syntenic block *FZD6-Cthrc1-slc25a32* is likely gnathostome specific, as it is not found in lamprey or in other chordate genomes (fig. 4*C*). The origin of the *FZD6* and *Cthrc1* genome linkage thus coincided with the decrease in sequence evolution of *Cthrc1*. Slc25a32 is present in most bilaterian and cnidarian genomes and, in contrast to *Cthrc1*, does not show marked difference in branch length between vertebrates and nonvertebrates (supplementary fig. S9, Supplementary Material online). FZD6 instead originated at the base of vertebrates from the duplication of *FZD3/6*, a gene which is still present in ascidians (supplementary fig. S10, Supplementary Material online).

Analysis of the NCBI databases shows several forms of the *Cthrc1* gene in vertebrates. In human, an alternative 5′ exon containing a translation initiation site is located downstream of the conserved first exon. This isoform is also predicted from the genomic sequence in hominids and in old world monkeys (e.g., baboons, mandrils, and gibbons), but not from other primates and other mammalian genomes (supplementary table S4, Supplementary Material online). We therefore hypothesize that it originated in the common ancestor of old world monkeys and apes (catarrhines) about 30 Ma (Kumar et al. 2017). The alternative N-terminal coding sequence does not contain a signal peptide and is thus not predicted to be secreted like most *Cthrc1* gene products. The exact phylogenetic distribution and possible function of this alternative isoform await future studies.

### Structural Characterization of the C-Terminal Domain of Cthrc1 Proteins

**Fig. 5.**
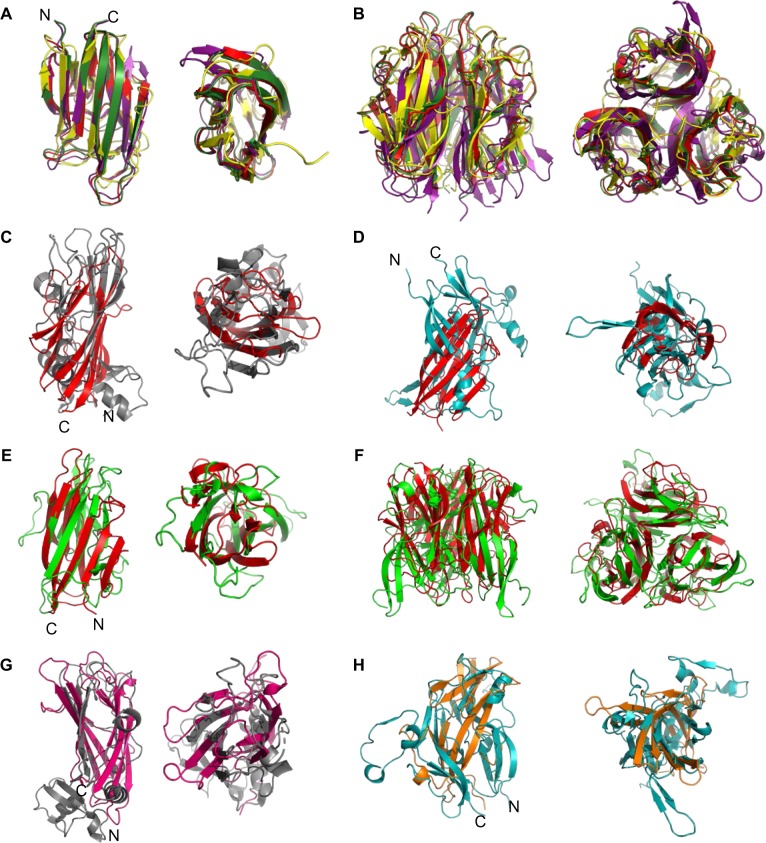
Comparisons of Cthrc1 Rosetta derived models and structural models of collagens and the C1q domain. Structural alignment of monomers (*A*) and trimers (*B*) of the C-terminal domains of collagen Alpha1(VIII) NC1 (red, PDB: 1o91), human collagen X NC1 (forest green, PDB: 1gr3), BclA collagen-like protein (yellow, PDB: 1wck, a top-scoring hit from HHpred search), and the globular head of the complement system protein C1q (purple, PDB: 1pk6). All the structures consist of a ten-stranded β-sandwich. The monomer alignment contains 80% overlap of matching Cα atom pairs (RMSD, root-mean-square deviation, 1.5 Å). (*C*) Monomer of collagen Alpha1(VIII) NC1 (red, PDB: 1o91) aligned to the fibrillar procollagen type III (gray, PDB: 4ae2). (*D*) Monomer of collagen Alpha1(VIII) NC1 (red, PDB: 1o91) aligned to collagen type IV (teal, PDB: 5nay). (*E*) Nve-Cthrc953 (v1g197953) monomer model (an eight-stranded β-sandwich, green) aligned to monomer of collagen Alpha1(VIII) NC1 (red, PDB: 1o91). (*F*) Trimer of v1g197953 (green) generated by alignment to collagen Alpha1(VIII) NC1 trimer (red). (*G*) Nve-Cthrc455 (v1g124455) monomer model (hot pink) versus the fibrillar procollagen type III (gray, PDB: 4ae2). (*H*) Nve-Cthrc666 (v1g216666) monomer model (orange) versus collagen type IV (teal, PDB: 5nay).

The Cthrc1 protein was predicted to contain a short collagen triple helix domain and was shown to be a secreted protein (Pyagay et al. 2005; Yamamoto et al. 2008), but no further structural information was available to date. Its C-terminal domain, encompassing more than half of its size, did not show homology to other proteins using BLAST searches. In order to identify proteins with 3D structures similar to the conserved C-terminal domain of *Cthrc1* genes, we performed a remote homology search using HHpred (Soding et al. 2005; Zimmermann et al. 2018). HHpred is among the most sensitive methods for the detection of remotely related sequences, representing the query sequence and the database proteins using hidden Markov models profiles and searching with profile–profile comparisons. When submitting the human CTHRC1 sequence as a query, the top-scoring hit was the mouse collagen Alpha1(VIII) C-terminal noncollagenous (NC1) domain (PDB: 1o91) (Kvansakul et al. 2003) (supplementary fig. S11, Supplementary Material online). Additional high scoring hits included the human collagen X NC1 (PDB 1gr3) and the BclA protein, a bacterial collagen-like protein (Yu et al. 2014) (PDB: 1wck), which is likely a product of horizontal transfer from animals to bacteria (Rasmussen et al. 2003). The same hits were detected for most *Nematostella Cthrc1* queries (e.g., *Nve-Cthrc953*, for which the hit with highest sequence identity [18%] was also the BclA protein, supplementary fig. S11, Supplementary Material online). In addition, for most queries, we also found matches to members of the complement C1q superfamily. When we superimposed the three NC1 structures of the top Cthrc1 hits, as well as that of C1q, we observed that they share the same basic β-sandwich fold with a “jelly roll” topology (reviewed by Kishore et al. [2004], fig. 5*A*) and that they form trimers (fig. 5*B*). HHpred queries with Cthrc1 from other species mostly returned similar results but occasionally gave other collagens such as collagen type IV and procollagen type III as the top hits but usually with much lower scores and shorter aligned regions. As could be expected, these collagens displayed different structural organizations upon alignment (fig. 5*C* and *D*).

In the HHpred searches using full-length Cthrc1 as queries, the sequence identity was high for the triple helix and low for the C-terminal domain (supplementary fig. S11, Supplementary Material online). When HHpred searches were performed with C-terminal domains only there were no significant hits. Thus, to test whether the Cthrc1 C-terminal domain can indeed form a β-sandwich fold as suggested by the full-length sequence HHpred analysis, we used the ab initio folding algorithms Rosetta (Simons et al. 1997) and I-TASSER (Yang, Yan, et al. 2015). These ab initio folding methods predict proteins tertiary structures from their primary amino acids sequences without relying on solved homologous structures. We obtained 1,000 best scoring models from Rosetta and 5 from the I-TASSER server. All the models contained one or two β-sheets, and the β-sandwich fold was present in many of them. Structural comparison to collagens VIII and X, previously found by HHpred, revealed a significant structural similarity (over 60% overlap, table 1) supporting the β-sandwich fold prediction for both the human and *Nematostella* (fig. 5*E*) Cthrc1 proteins. We also checked whether the trimer can be formed by superposition of the monomer models on the trimer structure. Indeed in all cases, the trimer can be assembled without significant steric clashes between the subunits (fig. 5*F*). Thus, our results demonstrate that the “orphan” C-terminal domain of *Cthrc1* genes is most similar in structure to the C-terminal NC1 domain of the types VIII and X collagens, the bacterial collagens and C1q, and less so to the C-terminal COLFI domain of the fibrillar procollagen type III and collagen type IV (table 1 and fig. 5*G* and *H*).

**Table 1:** Structural Comparison of Solved PDB Structures of C-Terminal Domains of Collagens and C1q to the Rosetta-Generated Models for Cthrc1 C-Terminal Domains

Collagen C-Terminal Structure	Cthrc1 Protein	Cα Overlap (%)	Quality of Alignment Measured by RMSD (Å)
Collagen Alpha1(VIII) (PDB: 1o91)	*Nematostella* Nve-Cthrc953	63	2.33
Collagen Alpha1(VIII) (PDB: 1o91)	CTHRC1 NP_612464.1	66	2.41
Fibrillar procollagen type III (PDB: 4ae2)	*Nematostella* Nve-Cthrc455	20	2.45
Collagen type IV (PDB: 5nay)	*Nematostella* Nve-Cthrc666	37	2.43

note.—The comparison shown is to the best scoring *Nematostella* Cthrc1 protein and to the human CTHRC1 for comparison. RMSD, root-mean-square deviation.

## Discussion

### Beyond the Tip of the Iceberg: *Cthrc1* Genes Are Found in Many Metazoan Groups

The bulk of the scientific literature on *Cthrc1* genes describes their involvement in an ever increasing number of human cancer types (Tang et al. 2006; Jiang et al. 2016). We set out to explore the phylogenetic distribution of Cthrc1. Presence of the Cthrc1 C-terminal domain in many metazoan clades, as well as in few choanoflagellates (fig. 3*A* and supplementary table S2, Supplementary Material online [Richter et al. 2018]), suggests that this domain was present in the last choanoflagellate-metazoan common ancestor. In choanoflagellates, the Cthrc1 C-terminal domain is found without the CTHR domain suggesting that Cthrc1 may represent a metazoan novelty in the fusion of two preexisting domains: the short collagen repeat domain and the C-terminal C1q-like domain. Such domain fusion is a known evolutionary mechanism generating novel proteins in metazoans (Kummerfeld and Teichmann 2005).

The presence of the Cthrc1 C-terminal domain in a few dinoflagellate and bacterial proteins questions the origin of this domain. Interestingly, we could find predicted proteins from bacterial metagenomes containing both CTHR and Cthrc1 C-terminal domains. We could nevertheless confidently conclude that the pairing between these two domains occurred independently in Bacteria and Metazoa, representing a case of *merology* or convergence of domain organization (Leclère and Rentzsch 2012). More data are needed to infer whether the Cthrc1 C-terminal domains found in bacteria and dinoflagellates were vertical inherited or originated from lateral gene transfer events. Our phylogenetic reconstructions supporting a grouping of bacteria and dinoflagellate Cthrc1 C-terminal domains, as well as the symbiotic nature of these organisms, are in favor of the latter hypothesis.

When did Cthrc1 appear in Metazoa by fusion of CTHR and Cthrc1 C-terminal domains? From their distribution in nonbilaterian animals, and considering the unsettled issue of the phylogenetic position of ctenophores, it is for now impossible to conclude whether Cthrc1 was present in the last common ancestor of Metazoa and whether it was lost in ctenophores. Under the hypothesis of sponges being the sister-group to all the other animals (Feuda et al. 2017; Simion et al. 2017), *Cthrc1* likely originated in the last common metazoan ancestor and was lost in ctenophores, whereas a later origin in the common branch of sponges and other nonctenophore animals would be favored under the ctenophore-sister hypothesis (Ryan et al. 2013; Whelan et al. 2017).

In spite of the uncertainty about the origin of Cthrc1, we could confidently infer many losses of this gene family during metazoan evolution as well as gene family expansion in other groups. Several of the groups that lost *Cthrc1*, such as nematodes, appendicularians, Platyhelminthes, and *Hydra*, are known for being prone to gene losses (Chapman et al. 2010; Denoeud et al. 2010; Mitreva et al. 2011). Conversely, several of the groups showing an expansion of *Cthrc1* genes—such as priapulids, anthozoans, hemichordates, and cephalochordates—are known for retaining a large number of gene families (Webster et al. 2006; Putnam et al. 2007; Simakov et al. 2015; Marletaz et al. 2018). The number of *Cthrc1* genes in a given group seems thus to be correlated with its genomic “plasticity.” Counter examples could however be found, both with more “plastic” groups retaining *Cthrc1* (e.g., ascidians and hydrozoans) and more “conservative” groups losing it (e.g., Mollusca and Placozoa). This phylogenetic pattern, nevertheless, suggests a rapid evolution of the function of *Cthrc1* in Metazoa.

The conserved structure of Cthrc1, together with our current knowledge of the molecular activity of these proteins in vertebrates, suggests a conserved signaling function across metazoans. Interestingly, many of the groups harboring multiple Cthrc1 display indirect development, with an intermediate larval-like form before the adult stage, and high regenerative capabilities. This would correlate with the epithelial–mesenchymal transition and migration promoting abilities of Cthrc1 shown in vertebrates (Tang et al. 2006; Hou et al. 2015; Ni et al. 2018) and the known interaction with TGFβ/BMP and Wnt signaling pathways (Pyagay et al. 2005; Tang et al. 2006; Yamamoto et al. 2008; Dong et al. 2018). Many of the regeneration model species (*Hydra*, planaria, *Hofstenia*, and *Botryllus*) have nevertheless lost *Cthrc1*. These highly regenerative animals, however, all rely on specialized stem cell populations, which might have disengaged them from the putative Cthrc1-dependent remodeling. Functional data on *Cthrc1* genes among different metazoan groups are required for a better understanding of the repeated diversifications and losses of this gene family and to assess its signaling function and contribution to body patterning and regeneration.

### High Conservation of the Vertebrate *Cthrc1* Genes

Following our phylogenetic analyses, we inferred that the vertebrate *Cthrc1* genes were remarkably conserved in sequence and structure, with identical size for the CTHR domain in all vertebrates, in contrast to the high variation observed between nonvertebrate groups (see fig. 4*C*). Most vertebrates harbor a single *Cthrc1* gene, which most likely represents the ancestral condition, and all of the vertebrate *Cthrc1* inferred gene duplications could be traced back to later genome duplication events (fig. 4*A*).

Although vertebrate *Cthrc1* genes are highly conserved and slowly evolving, the *Cthrc1* gene of the naked mole-rat shows a considerably faster evolution rate as compared with close groups (see fig. 4*A*). This might be of interest, as this species is known for its cancer-resistance and longevity (reviewed by Gorbunova et al. [2014]), and *Cthrc1* was shown to be upregulated in many cancer types in mammals and to contribute to cancer cell invasion and metastasis (reviewed by Jiang et al. [2016]).

A possible explanation for the slow evolution of the vertebrate *Cthrc1* gene lies in in its genomic environment, and its linkage to the *FZD6* gene. This gene was shown to be one of the Cthrc1 binding partners in mouse (Yamamoto et al. 2008) and to regulate the expression of *Cthrc1* (Dong et al. 2018). We can speculate that the genomic proximity between these two genes reflects a functionally important coregulatory expression mechanism that has evolved in gnathostomes, which may have led to the “stabilization” of *Cthrc1* sequence and copy number in contrast to other metazoan groups. Whether the conserved genomic colocalization between *Cthrc1* and *FZD6* is indeed necessary for the functional interaction of these genes remains an open question, which should be addressed by comparing the mode of regulation of these genes in several vertebrate species. The question also remains as to the origin of the *Cthrc1-FZD6* genomic linkage. Due to the incomplete genome assembly status and the conflicting results for the phylogenetic position of the *FZD3*/*FZD6*-like genes of lamprey and hagfish (supplementary fig. S10, Supplementary Material online), we could not conclude whether FZD6 originated before or after the common ancestor of vertebrates. New genomic resources for hagfish and lampreys will allow establishing whether the *FZD6*-*Cthrc1* linkage is an ancestral trait of vertebrates or gnathostomes.

Functional knowledge about *Cthrc1* is limited and restricted mostly to mouse where knockout of this gene did not lead to decreased viability or compromised reproduction, but rather to metabolic defects mainly in muscle and adipose tissues (Yamamoto et al. 2008; Stohn et al. 2012, 2015). Recent work described an essential role for *Cthrc1b*, one of the two zebrafish paralogs, in the epiboly and convergent-extension cell movements during gastrulation. This work further showed that during gastrulation Cthrc1b promotes integrin-mediated cell adhesion (Cheng et al. 2019). Whether this early function of Cthrc1 is common among vertebrates, or specific to some fish groups harboring multiple *Cthrc1* genes, warrants further research. A deeper functional characterization of Cthrc1 in different vertebrate models—in particular *Cthrc1* KO adult mutant phenotypes and overexpression experiments—would allow better understanding of the slow evolution of Cthrc1 among vertebrates.

### Structural Definition for the Cthrc1 “Orphan” C-Terminal Domain

Our structural analyses showed that the hitherto “orphan” C-terminal domain of Cthrc1 proteins bears similarity to collagen and collagen-like protein domains for which structural data exist. We therefore concluded that the C-terminal domain of Cthrc1 shows the highest similarity to the globular C1q domain of collagens VIII and X and less to the COLFI domain of fibrillar collagens and to the C-terminal domain of collagen IV. In the original description of *Cthrc1*, the authors noticed the overall similarity with proteins containing a short collagen domain (i.e., CTHR) and a C1q/TNF domain (Pyagay et al. 2005). They, however, did not find sequence homology, as is indeed the case without using remote homology search. The globular C1q domain is found in proteins containing a CTHR domain (e.g., collagens VIII and X, complement C1q), as well as in many others that do not contain a CTHR domain (e.g., Cerebellin, Caprin, and TNF) (Kishore et al. 2004; Carland and Gerwick 2010). The association between C1q and CTHR domains, as present in collagen VIII/X and complement C1q proteins, is thought to be chordate specific, whereas the C1q domain can be found in many other metazoan groups as well as in bacteria (Carland and Gerwick 2010). The high structural similarity of Cthrc1 to the network forming collagens VIII and X is thus most likely coincidental and due to their shared CTHR–C1q-like domain composition.

Is Cthrc1 a bona fide collagen? The term collagen refers to a diversity of extracellular matrix protein families all sharing a CTHR domain (reviewed by Ricard-Blum [2011], Hynes [2012], and Fidler et al. [2017, 2018]). The different collagen types form diverse supramolecular structures in the extracellular matrix of many cell types, including long fibrils for strength and mechanical force of multiple tissues (e.g., collagen types I–III) and basement membrane networks for the support of epithelial cells (collagen IV). Several domains are found at the C-termini of collagens, such as the C1q-like of type VIII and X collagen, the COLFI domain of fibrillar collagens and the C4 domain of type IV collagens. Most collagens contain a very long CTHR domain composed of hundreds of GXY repeats and thus differ markedly from Cthrc1 displaying a short CTHR domain. The most ancient collagen type is considered to be the network forming collagen IV, a variant of which first appeared in a single celled common ancestor of filastereans, choanoflagellates, and metazoans (Grau-Bove et al. 2017; Fidler et al. 2018). Fibrillar collagens are metazoan specific (Rodriguez-Pascual and Slatter 2016), whereas collagens VIII and X are found only in Chordates (Fidler et al. 2018). We propose that Cthrc1 is a collagen-like protein whose C1q-like C-terminal domain, found in both metazoans and choanoflagellates, fused with a short CTHR domain early during animal evolution. This fusion product led to a new signaling protein family, which is overall similar in its general mode of molecular activity to other C1q family proteins in contrast with a structural role typical to “classical” collagens. We showed that *Cthrc1* was retained and duplicated in several animal clades, lost from many others, and was highly conserved in terms of sequence, domain size, and copy number in the vertebrate lineage.

## Supplementary Material

evaa020_Supplementary_Data
