# Supplementary material for: Dynamic Evolution of the Cthrc1 Genes, a Newly Defined Collagen-Like Family
Source: Genome Biol Evol. 2020 Feb 8;12(2):3957–70. doi: 10.1093/gbe/evaa020 (PMC7058181; doi:10.1093/gbe/evaa020)
Supplement: evaa020_Supplementary_Data [file evaa020_supplementary_data.zip › Leclere-et-al-Supp-Tables-Figures.pdf]

SUPPLEMENTARY MATERIAL

Dynamic evolution of the *Cthrc1* genes, a newly defined collagen-like family

Lucas Leclère, Tal S. Nir, Michael Bazarsky, Merav Braitbard, Dina Schneidman-Duhovny and Uri Gat

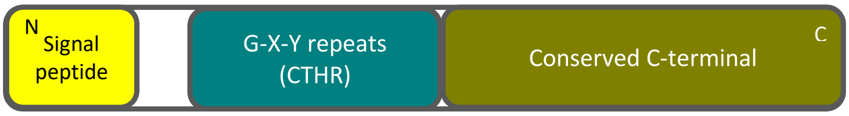


```
Nve-Cthrc455        ----------MMNFSCFCLLWVVMLINIVSCQREKGQRPTPMFWGIDNCNKLEENCGMRF
Nve-Cthrc086        ----------MMKYLLIVSLVTALAMTAMTQDVRRVLNSDDGKSSNPDDQSKTKQC----
CTHRC1              MRPQGPAASPQRLRGLLLLLLLQLPA---------PSSASEIPKGKQKAQLRQREV----
Nve-Cthrc666        -----------MKLSLVFVGVVQLSLIL-------AHTSSRC-----------KNC----
Nve-Cthrc415        ----------MLKFLLIFVGAVQLSLIL-------AHAPSPC-----------QNC----
Nve-Cthrc953        ---------MSIILSMIILGVV--------------------------------------
Nve-Cthrc153        --------MKVILSTCIFLL----------------------------------------
Nve-Cthrc537(1)     ----------MQLLLLVFLCICGLSWGQI------DWGPGAC-------AQMAKACWPLP
Nve-Cthrc537(2)     ----------MKIYEIFFVISVTILCR--------NAVSVRSQNIDWRCSSMLQSC-ELN
                                    .

NveCthrc455         QGSKGTPGAPGDPGLYGPPGPPGKKGPKGPKGRTAILPNKAMIGAIPPFYGPKGDRGVPG
NveCthrc086         -----------TPCSCGMPGIPGTHGIPGTPGPAGIAGLPGPKGDRGN----PGRRGQWG
CTHRC1              -----------VDLYNGM----CLQGPAGVPGRD--------------------------
NveCthrc666         -------CTHGHDGKNGVPGIPAIPGKPGTPGNS--------------------------
NveCthrc415         -------CAQGHDGKNGIPGIPGVHGKPGTPGKS--------------------------
NveCthrc953         -----------------MRGGHATPGTDTKLSKH--------------------------
NveCthrc153         ----------------GLGYVANQPGPDTVIAKH--------------------------
NveCthrc537(1)      KGAQGPPGPPGEAGPAGRRGAPGPPGPPGPRGYPGRVLTPPPGPPAP-----KGDRGEPG
NveCthrc537(2)      NGIIGLKGNQGFPGPMGLDGIPGIPGPPGDRGDPG---TSPRVPPFPYWHRVKGAKGEIG
                                             *     .

Nve-Cthrc455        RQGMYGLRGPPGRKGDQGTRGPPGLNGPTGAQGDSGFPGVPGPKGFPGGIGSVR-SWKSC
Nve-Cthrc086        PVGPKGDQGSPGVWGKPGKAGLPGKTGPRGLPGESGLPGIRGPKGEPGPNARRQATWKQC
CTHRC1              --------------GSPGANGIPGTPGIPGRD---GFKGEKGECLRESFEESWTPNYKQC
Nve-Cthrc666        --------------GDVGRDGRPGAPGPKGPP---GPKGSRGPMGVQG--GTTHVNWKQC
Nve-Cthrc415        --------------GNDGRNGKPGAPGPKGPP---GPKGSRGPKGVQGPPGTTHVNWKQC
Nve-Cthrc953        --------------GFGPLTFIAQAPGPRGPP---GSPGTPA-----------HVNWKQC
Nve-Cthrc153        --------------SFGPITFIAQAPGPAGPA---GPPGTPA-----------HVNWKQC
Nve-Cthrc537(1)     YPGEPGEGGDVGFPGERGLRGFGGFPGAKGHK---GKEGDTGLPGRRGPPGSSSSNAREC
Nve-Cthrc537(2)     FPGIKGEKGLKGFPGTPGDQGRQGVPGKRGYDGAKGREGEDGANGPPGSPGSANMNWKQC
                                  .           *  *     *  *  .             . ..*

Nve-Cthrc455        TWKF-DPTQEYGLVKECDFVKHHSATLLRVTYEGSMRTGFCTK--CCKRWYFTFNLEECS
Nve-Cthrc086        TWKR-SDSMAEGKIQECFFTKEEANTSIKVSFHGNIQVSGRRG--ACNRWYFKINDRECS
CTHRC1              SWSSLNYGIDLGKIAECTFTKMRSNSALRVLFSGSLRLKCRNA--CCQRWYFTFNGAECS
Nve-Cthrc666        AWKK-EDARDHGLLKDCVFRKRHQNTSLRVQFVGTLRIYNCNG--CCKRWFFTFNGAECN
Nve-Cthrc415        AWKK-SDERDNGLIKDCVFRKRRQNTALRVQFVGTFRIHNCNT--CCKRWFFTFNGAECR
Nve-Cthrc953        VWTNINEGKDVGLIKECAFNKHSDSTALRVAFDGDYRVATCVGVTCCRRWYFTFNHHECK
Nve-Cthrc153        AWKNLNDGKDSGLIKECVFNKLNDSTSLRVAFDGEFRVAFCQQ--CCRRWYITFNQNECS
Nve-Cthrc537(1)     MWKM-DDGKDRGVIVNCTIQKTSSSTAIHVTYNGPIEIGLCKR--CCKRWFFTFNGQECR
Nve-Cthrc537(2)     VFEL-ELNKQNGIIYECRFLKYSNVTSLRISYHGDTQIGLCGS--CCKHWRFLLDGQPCW
                     :   .     * : :* : *    : :.: : *           .*..* : ::   *

Nve-Cthrc455        APHRIEAR---L---EGLK--DHPFPEYRHDQLDGQCV--LPQGRVNVGLWVEDC--TGH
Nve-Cthrc086        SPETIEAL---YYYEWLSN--STPPAMRYHRTLEGYCG-GIPRGSVRVELWVGSC--PGE
CTHRC1              GPLPIEAI---IYLDQGSPEMNSTINIHRTSSVEGLCE-GIGAGLVDVAIWVGTC--SDY
Nve-Cthrc666        GPLTIEGV---IYMDKGTG--ANIQNLHRVRVIKGYCT-NIHKGTIRVGFNVGNC--AGY
Nve-Cthrc415        GPLPIDGV---LYMYKGSG--ANLQELHRVRVIEGYCT-NIHKGTIRVGFNVGNC--AGY
Nve-Cthrc953        HPAPIDAA---QF--HGGD--ISGHNVHEHRHVEGYCQ-GIAKGQVRVGFWVDSCKFSRY
Nve-Cthrc153        NPVPIDAV---QH--PSIV--VANFNLHEHRHFGGYCE-GIPRGQVRVGLWVGQC--VGT
Nve-Cthrc537(1)     SPAPIDAI---M---SGGL--RPDYPHYQYGRVEGICQVPFPAGYLRVSLNLENC--KGF
Nve-Cthrc537(2)     SPGPIDATYSGLMLVNVNQ--RNTLPVFRHGSISGYCN-SLPRGVVRVALDLSNC--PGY
                     *  *:.                         . * *   :  * : * : :  *

Nve-Cthrc455        TRSPKSN---ITLVDMSGRMFIEEVDVVQSNNRI
Nve-Cthrc086        --KPGYG---RVGWRSVSRMIIEEVTPPQV----
CTHRC1              --PKGDA---STGWNSVSRIIIEELPK-------
Nve-Cthrc666        --GNADA---HTGWNAVSRIIVEEVPPQQS----
Nve-Cthrc415        --GNADA---HTGWNAVSRIIVEEVPPQQT----
Nve-Cthrc953        HDGPADA---YTGWMSSTRIVIEEVPGPQA----
Nve-Cthrc153        NMGNADA---STGWMSSTRIIVEEVPAPQA----
Nve-Cthrc537(1)     --QDSDVPIKVSLYKPNGLVTLHEIVPPQ-----
Nve-Cthrc537(2)     ---PSDSIVVSTEVVATGRIIIEEVPAPQA----
                                       : : *:
```

Figure S1: Multiple sequence alignment of the human CTHRC1 (isoform 1) and the eight Nve-Cthrc protein sequences. The alignment was done using MUSCLE (Edgar, R.C. 2004 BMC Bioinformatics 5:113) and color-marked manually according to the domains (as depicted in the protein scheme) and conserved positions. The “A” marked in red in the CTHR domain of Nve-Cthrc666 was found to be G in several cDNA sequencing operations. The KGD marked in yellow in the C-terminal CTHRC1 sequence is a putative conserved integrin binding motif according to Cheng et al., 2019. Markings: (*) positions which have a single, fully conserved residue; (:) conservation between groups of strongly similar properties; (.) conservation between groups of weakly similar properties.

```
CTHRC1               --------MRPQGPAASPQRLRGLLLLL--------LLQ-----------------LPAP
Che-Cthrc001541      --------------MKIFHLFVYLFLLF-------NYHQCKHSEDQSTVDDIENDVLMSK
Che-Cthrc010641      ------------MARIMMAVTVVLFLLFCNKFIVGDKADKDEFKQDDIVMKDNSSQGSKP
Che-Cthrc016334      --------------MKKIIFFIQILVVLGSTSSISEFGNEEAVLDAKSSQKKTLA-ESDS
Che-Cthrc020578      --------------MKKVILFIQILLVL-GSTSSTDVANEDAALDTKSSPKKTLA-QSSP
Che-Cthrc023343      --------------MKKVILFIQILLVLGSTSSTSEVENKEASLDTKSSPKKTLA-QSSP
Che-Cthrc031718      -------------MAICSLFLAGLLISF--------IHGDVTIKETETLGYNLKDSSSSN
Che-Cthrc033649      ---------------MDFKIKLAIFLLS----------------------------LSTI
Che-Cthrc013082      ---MHLRIQMSKVRGNYPLNHKAVLRLQWLRLNHQDIQNRESQF------------QVKG
Che-Cthrc039542      ------------------------------------------------------------
Che-Cthrc037910      --------------MVKGFVAFRILLVF--------VVAVSCVC------------SEKQ
Che-Cthrc013079      -----------MNKLICIPVLLTMMFVV-----------------------------VST
Che-Cthrc024151      -----------MNKLICIPVLLAMMFVF-----------------------------VST
Che-Cthrc033869      ---------------MTMKTVVFILLVT--------VLACTQ--------------AMKN
Che-Cthrc013076      ---------------MKTVLTFFLLFVA--------VLGCIQ--------------ATKN

CTHRC1               SSASEI------PKGKQKAQLRQ----------------------------------REV
Che-Cthrc001541      SRHYFCKNVLQNRDGKDGRDGRDGRDGIRGPPGEKGSTGRAGKDCDQQMQWERIQHRPPS
Che-Cthrc010641      STCGAC------HGGRDGRDGHPG---------------------------------HPG
Che-Cthrc016334      STCQPC------RKGRDGRDGLNG---------------------------------LPG
Che-Cthrc020578      STCQPC------RKGRDGRDGLNG---------------------------------LPG
Che-Cthrc023343      STCQPC------RKGRDGRDGRDGLNG------------------------------LPG
Che-Cthrc031718      SRCSVC------RHGRDGLNGRD-------------------------------------
Che-Cthrc033649      SCHSQC------CKGKDGKDGRD-------------------------------------
Che-Cthrc013082      ECNSSC------KVGRDGRDGRNGIDGKDGKQGPAGKPGKNGADGIDG---------ING
Che-Cthrc039542      -----------GRDGRDGIDGKDGKQGPAEKPGKNGADGIDG---------------ING
Che-Cthrc037910      ECRARC------KDGRDGRNGRNGVDG------------------------------VDG
Che-Cthrc013079      ANNQEC------KDGRDGRDGKD------------------------------------G
Che-Cthrc024151      ANNQEC------KDGRDGRDGKDG---------------------------------KDG
Che-Cthrc033869      DNNGLC------KDGRDGRDGKDGKDGI-------------GLNGKDG---------KDG
Che-Cthrc013076      DCSNCC------KDGRDGRDGKDGI----------------GKDGKDGIA-------ING
                                   *.:  :

CTHRC1               VDLYNGMC-------------------------------LQGPAGVPGRDGSPGANGIPG
Che-Cthrc001541      NDGTQGLTGEKGDKGAPGKDGAHGTPGLPGVPGRDGINGTIGEKGSPGLPGRDGENGTAG
Che-Cthrc010641      RDGRDGCP------------------GALGPKGQAGVNGLPGTDGKNGINGKPGKNGSQG
Che-Cthrc016334      RDGEDGCPGLAGRDGLP---------GATGPEGPQGIQGRPGSNGTKGDQGVPGKDGKNG
Che-Cthrc020578      RDGEDGCQGQAGRDGLP---------GARGPEGVQGIQGRPGSNGTKGDQGVPGKDGNNG
Che-Cthrc023343      RDGEDGCQGPAGRDGLL---------GVRGPEGVQGIQGRPGSNGTKGEQGVPGKDGKDG
Che-Cthrc031718      -----------------------------------------GVNGRDGVNGIDGCDGMPG
Che-Cthrc033649      ------------------------------------------------------------
Che-Cthrc013082      VDGKDGKDGIHGKDGKDCQMGMRGRDGLNGTNGKDGMNGKDGKDSLNGIDGMDGLNGTNG
Che-Cthrc039542      VDGKEGKDGIHGKDGKDCKMGMRGRDGLNGTNGKDGMDGKDGKDGLNGIDGKDGLNGTNG
Che-Cthrc037910      RNGIDGFC---------------------------------GKHGINGTNGIDGKDGRNG
Che-Cthrc013079      RDGKDGVC-----------------------LYKKGVFGIKGEKGEDGKDGINGKNGTNG
Che-Cthrc024151      KDGKDGVC-----------------------LYKKGVFGIKGEKGEDGKDGINGKDGTNG
Che-Cthrc033869      RDGRNGINGKDGKDGKQGLNGKPGKDGLNGKNGKDGLNGKPGEDGKNGIDGRDGSNGAPG
Che-Cthrc013076      KDGRDGRNGVDGKNGINGKPGKDEKDGIDGKDGFKGTNGEDGKNGEPGKDGKDGIDGKDG

CTHRC1               TPGIP---------------------------------GRDGFKGEKGECLRESFE----
Che-Cthrc001541      IQGPP---------------------------------GESGRNGTDGKPGKNGAPGIPG
Che-Cthrc010641      LPGKD---------------------------------GADGKPGKNGKNGINGK-----
Che-Cthrc016334      INGAP---------------------------------GVNGKPGLPGPPGKDG------
Che-Cthrc020578      INGVP---------------------------------GVNGTKGEQGPPGKDG------
Che-Cthrc023343      INGVP---------------------------------GVNGRPGPQGRPGKDG------
Che-Cthrc031718      KQGVQ---------------------------------GPPGKDGLQGSTGKDGK-----
Che-Cthrc033649      --GRD---------------------------------GRDGKDGESCSVSEVQ------
Che-Cthrc013082      RDGIDGIDGKDGLNGLNGIDGMDGLNGTNGRDGIDGIDGKDGLNGLNGIDGLDGINGANG
Che-Cthrc039542      RDGVD---------------------------------GRDGLNDSNGIDGLDGINGANG
Che-Cthrc037910      KDGKD---------------------------------GRNGKDGKNGRDGRDGKD----
Che-Cthrc013079      KDGIN---------------------------------GKDGKDGRDGKDGKDG------
Che-Cthrc024151      KDGIN---------------------------------GKDGNDGKNGKDGKDG------
Che-Cthrc033869      KDGLN---------------------------------GKDGKDGIDGKDGNNGN-----
Che-Cthrc013076      KNGID---------------------------------GKDGKDGKDGKDGKHGTN----
                       *                                   *  *  .

CTHRC1               ----ESWTPNYKQCSWSSLN----YGIDLGKIAECTFTKMRSNSALRVLFSGSLRLKCRN
Che-Cthrc001541      EDGNPGRVGNWKECKWNNIQ----DSRTRGLIKECSFVKKYPHTYVKVTVVTNVHIRSNT
Che-Cthrc010641      ----DRTARNIKQCAWKRLSTNNYDTTEIGLIKECSIQKRVASSELLVTVSTTIRVYSCK
Che-Cthrc016334      ----SIGGRNFKQCAWDFTS-----SKDSGLLKACIFTKSKTDSYLMVTISSNVYQGKAR
Che-Cthrc020578      ----SIGGRHFKECAWRFTS-----STDTGLLKDCKLNKSKTDSYLMVSISSNIYQGKAQ
Che-Cthrc023343      ----SIGGSYFKECAWRFTS-----DKETGLLKDCKFNKSQTDSYLMVSISSNIRQGKNK
Che-Cthrc031718      ----DLLASQWKDCVWNHIN----DDKDKGLVRECSFKKKYATTYLRISVSSNGRVYGCS
Che-Cthrc033649      ----QVVEKNWKECAWNRIE----DGRDYGLIKSCAFNKKSNSSYLRVAVAGDMRLHNCN
Che-Cthrc013082      MNGKDSPKRNWKECAWDKSN----SELDAGLLKTCTFRKKSIETYLRVEVISNLRIYNCD
Che-Cthrc039542      MNGKDSPKRHWKECAWDKSF---YSELDAGLLKICTFRKKSIETYLRVEVISNLRIWNCD
Che-Cthrc037910      ----ITRKRNWKECAWDNIS----DGRDNGLIKTCSFSKSEQSTYLYVQVSSNMRIVNCD
Che-Cthrc013079      ------LTKNWKECAWNKID----DHKDNGVIKSCAFKRESPSTYLKVVVSSNMRIYNCN
Che-Cthrc024151      -------AKNWKECAWNKIN----DNKDNGVIKSCAFKKKSSSTYLKVVISSDMRIYNCN
Che-Cthrc033869      ----EPSKKNWKECAWNHIN----DGKDSGEIKHCLFKKNSIKSYLKVVASSNMRIVNCN
Che-Cthrc013076      ----ELLKKNWKECAWKNVN----DGKDNGVIKHCSFKKKSPSTYLKVFVSSNMRINNCN
                                *:* *             * :  * : .    : : :
```

```
CTHRC1              ACCQRWYFTFNGAECSGPLPIEAIIYLDQGSPEMNSTINIHRTSSVEGLCEGIGAGLVDV
Che-Cthrc001541     G--KRWFVTFDNKECSHQTPIGLFVSNVTG-----------FTPFYLTGSCLVNKTGIVLI
Che-Cthrc010641     NCCKRWFVTFDGDECA-PVPIDHALYRYEASR-------DFIPLTLKGLCKVNKSGLVRV
Che-Cthrc016334     A-CSRWFVTFDGKECS-PHPIDHVFY--RGY----KGQSEYIPFTMNGVCKINKSGIVSV
Che-Cthrc020578     A-CSRWFVTFDGKECS-PHPIEHAYY--RGTT--SNANGDHIPFTMNGVCKINKSGVVSV
Che-Cthrc023343     A-CSRWFVTFDGNECS-PYPIDHLYYR-------STANDDYIPFTLKGVCKINKSGVVSV
Che-Cthrc031718     GCCKRWFVTIDNKECA-PAPIDFSHYRAD-----AKTDDDVGQFLLKGNCRIPKTGSVKI
Che-Cthrc033649     NCCKRWFVTFDDKECS-PIPIDGIVYLMIGKG----TQNTHRPRVIRGHCKIPKRGAVNV
Che-Cthrc013082     DCCSRWFVTFNEQECT-PVPIDGIVYMKFGKG--DREKNLHRPQVITGRCKISEIGNINV
Che-Cthrc039542     DCCSRWFVTFNGQECT-PVPIDGTVYMKFGKG--NREKNLHRPQVITGRCKISKIGYINV
Che-Cthrc037910     ACCKRWFITFDGKECT-PVAIDGVVYMYKGKG--SRYKNLHRPRVIAGHCKINKSGRVNV
Che-Cthrc013079     ACCKRWFVTFDGHECA-PVPIDGVVFMREGTG--NRIKDLHRPRVITGHCKVSKKRQVNV
Che-Cthrc024151     ACCQRWFVTFDGHECA-PVPIDGIVYMGAGAG--NRLQNLHRPRVVRGHCKVSKKKQVNV
Che-Cthrc033869     SCCKRWFVTFDGHECA-SVPIDGVVYMDLGTG--SRQKNLHRPRVVRGHCKISKNHQVKV
Che-Cthrc013076     GCCKRWFVTFDGHECA-PVPIDGVVYMRAGAG--NRLKDLHRPRVIAGHCKVSKK-EVNV
                       .**:.*::  **:   .*                        : * *       : :

CTHRC1              AIWVGTCSDYP-K-GDASTG-WNSVSRIIIEELPK-------
Che-Cthrc001541     GFHVQNGS------VVAYTGHGNSSTNIIIEEVIPPQA----
Che-Cthrc010641     GLNIEDCGSKT-GSVDGDTGYYRSTFRILIEEVEPQS-----
Che-Cthrc016334     GFNVGKCKAIT-NIGDAYTG-FRSVTRIVIEEL---------
Che-Cthrc020578     GFNVGKCSGFD-SLGDAYTG-FNTVTRIVVEEL---------
Che-Cthrc023343     GFNVGKCSGFD-SLGDVYTG-WNQVTRIVIEEL---------
Che-Cthrc031718     GFHIGNCHGLG-D-ADGYTG-HKQVTRIMIEEIAPPQA----
Che-Cthrc033649     SINVGDCPDTG-P-GDAYTG-TQSSFRIFIEEVNAPQP----
Che-Cthrc013082     GFNIGECAGRN-T-ADASTG-WNQATRIYVEEIEGPQSDLIS
Che-Cthrc039542     GFNVGECAGAGKNTADAFTG-WQQATRIYVEEMEGPQSDLVS
Che-Cthrc037910     GFNVGNCRSYP-P-GDAHTG-WNSATRIHIYEMEPPQQ----
Che-Cthrc013079     ALNIGNCANTP-G-GDAHTG-WNSATRIYIEEVDQPQS----
Che-Cthrc024151     ALNVGNCTGFP-G-GDASTG-WNSATRIYIEEVDQPQS----
Che-Cthrc033869     ALNVGNCHGYG-N-ADAYTG-WNSATRIYIEEVDEPQS----
Che-Cthrc013076     ALNVGNCHGYG-N-ADAYTG-WNSATRISIEEVDPPQS----
                    .:.:              **  .   .* : *:
```

Figure S2: **Multiple sequence alignment of the human CTHRC1 (isoform 1) and the fourteen *Clytia* Che-Cthrc protein sequences.** The alignment was done using MUSCLE and color-marked manually according to the domains (as marked in the protein scheme) and conserved positions. Markings: (*) positions which have a single, fully conserved residue; (:) conservation between groups of strongly similar properties; (.) conservation between groups of weakly similar properties. * The predicted sequence of the Che-Cthrc010641 protein starts with NNSIKKTSSQTK before the M (and predicted signal sequence), hence it was considered mistaken and deleted from the alignment. ** No signal sequence was found for Che-Cthrc013082 and Che-Cthrc039542; alternatively, the N-terminus might not be complete. The putative integrin binding motif KGD is marked in yellow.

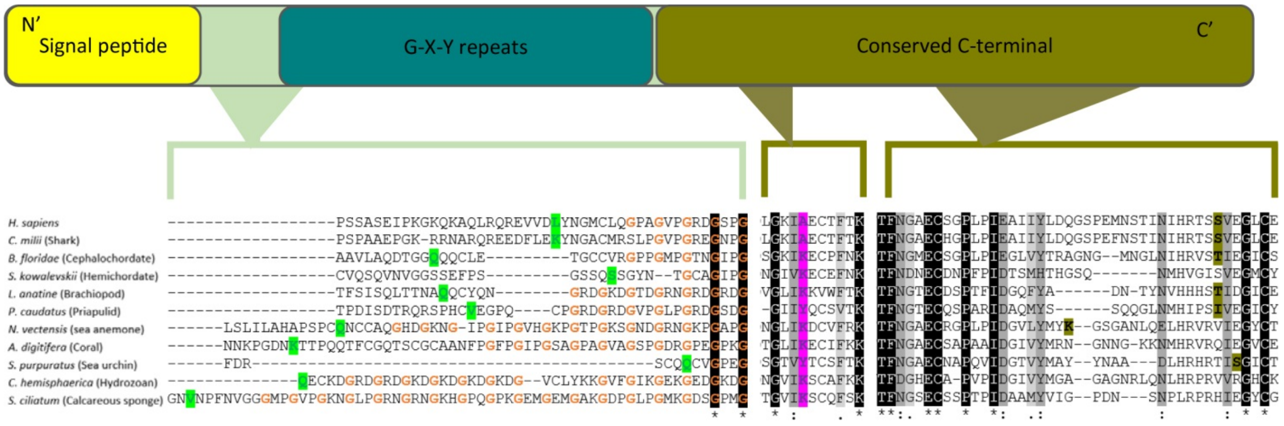


```
Homo sapiens                  --MRPQGPAASPQRLRGLLLLLLLQLPA-------------------------------
Callorhinchus milii           -----------MGCVAVLVLLTCLVLPS-------------------------------

Branchiostoma floridae        ----------MATIGRTHFLLGFLWLWS-------------------------------
Saccoglossus kowalevskii      --------MASFQIITIMFIVSTIVMLQ-------------------------------
Lingula anatina               ---------------MKYSTTVIFVLLA-------------------------------
Priapulus caudatus            ----------MQGIIIGVYLLSLGVCLV-------------------------------
Nematostella vectensis        ---------------MLKFLLIFVGAVQ-------------------------------
Acropora digitifera           MMAKRDISVPRLCAQLISFIALCSHANG-------------------------------
Strongylocentrotus purpuratus --------MVEIRWHRFVCLLLLALL-------------------------------
Clytia hemisphaerica          -----MNKLICIPVLLAMMFVFVSTANN-------------------------------
Sycon ciliatum                -----MRYSVLVACLLGSYLSTCSGQDRNVIETENVAPVTDDLPPYIREMFGNIKGLEV

H. ------------------PSSASEIPKGKQKAQLRQREVVDLYNGMCLQGPAGVPGRDGSPGANGIPGTPG-IPGRDGFKGEKG
C. ------------------PSPAAEPGK-RRNARQREEDFLEKYNGACMRSLPGVPGREGNPGTNGIPGTPG-IPGRDGIKGEKG
B. ------------------AAVLAQDTGGQQQCLE---------TGCCVRGPPGMPGTNGIPGGNGVPGSPG-ISGKDGLKGDRG
S. ------------------CVQSQVNVGGSSEFPS---------GSSQSSGYN--TGCAGIPGIPGSHGNPG-RDGRDGTKGERG
L. ------------------TFSISQLTTNAQQCYQN--------GRDGKDGTDGRNGRDGRDGLRGETGPPG----------PKG
P. ------------------TPDISDTRQRSPHCVEGPQ----CPGRDGRDGVPGLPGRDGSDGHPGAKGDKGDRGGNHGLSSEKG
N. ------LSLILAHAPSPCQNCCAQGHDGKNG-IPGIPGVHGKPGTPGKSGNDGRNGKPGAPGPKGPPGPKG-SRGPKGVQGPPG
A. ------NNKPGDNKTTPQQTFCGQTSCGCAANFPGFPGIPGSAGPAGVAGSPGDRGPEGPKGPQGSKGDEG-ARGRRGVQGPKG
S. ------FDR-------------------------------------------SCQQCVGPEGPNGPPGSEG-SQGTGTGDSWYD
C. --------------QECKDGRDGRDGKDGKDGKDGKDG--VCLYKKGVFGIKGEKGEDGKDGINGKDGTNG-KDGINGKDGNDG
S. GNVNPFNVGGGMPGVPGKNGLPGRNGRNGKHGPQGPKGEMGEMGAKGDPGLPGMKGDSGPMGPAGPEGMKG-DQGDFGMPGRRG
                                                              *  *  *  *  *            .

H. ECLR------------------------ESFEESWTPNYKQCSWSSLNYGIDLGKIAECTFTKMRSNSALRVLFSGSLRLKCRN
C. ECLR------------------------EILEEPWKINYKQCAWNSLNYGIDLGKIAECTFTKTRTDSALRVLFTGSLRLKCKS
B. KPGRPGKPGTKGLKGE-----------PGSSAALRAPNIKQCAWRKLSDDRDSGKIKECPFNKRASNSSLRVLWNGALRVTCPT
S. ETGAVGPPG---------------------IAELPVQNIKQCAWQNINDGRDNGIVKECEFNKMSDNSVLRVVWNGNLRAVSAS
L. EAGTP----------------------------CSPRNVKQFTWTDINDDRDVGLIKKVWFTKSTGTSMLRVGFHGNIRVMATA
P. EKGNAGINGI-----------------TPECPACEARKIHQCTWDSGN-----GIIYQCSVTKLRADTGLFVRWTGNLRNIRNE
N. TTHVN---------------------------------WKQCAWK-KSDERDNGLIKDCVFRKRRQNTALRVQFVGTFRIHNCN
A. ATGLSGPTGARGGRGEKGARGPQGSPGPKGADGLLVKNWKQCVYKNLNDGRDTGLIKECIFKKNSAKTGLRVFYNGDLRLHNCH
S. VMGSN-------------------------------RNWRQCTFN-WADGRDSGTVYTCSFTKQSAASSLYAAFSGTTRLHNCH
C. KNGKDGKDGAKN--------------------------WKECAWNKINDNKDNGVIKSCAFKKKSSSTYLKVVISSDMRIYNCN
S. MPGAQGPYGPKGEPGDTAAHPQ--------------ALWKQCAWTEES-NINTGVIKSCQFSKTSRDTHLRVAFGGNLRVGGCS
                                          ::  :         * :    . *    : * .   .  *

H. AC---CQRWYFTFNGAECSGPLPIEAIIYLDQGSPEMNSTINIHRTSSVEGLCEGIGAGLVDVAIWVGTCSD-YPKGD-ASTGW
C. AC---CQRWYFTFNGAECHGPLPIEAIIYLDQGSPEFNSTINIHRTSSVEGLCEGITAGLVDVSVWVGTCSDGYPQGD-ASTGW
B. GC---CKRWYFTFNGMECSGPLPIEGLVYTRAGNG--MNGLNIHRVSTIEGICSGLPQGRVNVELSVRNCDGISHSGN-AYTGW
S. GS---CTRWYFTFNDNECDNPFPIDTSMHTHGSQ-------NMHVGISVEGMCYGIGKGEIRVAFHVSTPCSGYPASD-AYTGW
L. GKSVACRRYWFTFNGTECDSPTFIDGQFYA------DN-TYNVHHHSTIDGICENLAAGEVVIGFHVGQCRILASETANAYTGW
P. DR--SCSRWYITFNGTECQSPARIDAQMYS------SQQGLNMHIPSIVEGICYELPTGRADIQFNVGACRNPAHKHGDSSTGW
N. TC---CKRWFFTFNGAECRGPLPIDGVLYMYK--GSGANLQELHRVRVIEGYCTNIHKGTIRVGFNVGNCAG--YGNADAHTGW
A. DC---CRRWYFTFNGAECSAPAAIDGIVYMRN--GNNG-KKNMHRVRQIEGVCEKIHKGIVRVGFWVGNCVG--YGTADAFTGW
S. GC---CCRWYLTFNGAECNAPQVIDGTVYMAY--YNAA---DLHRHRTISGICTNIAAGSVTVALVVGACSG--YGYFDCYTGW
C. AC---CQRWFVTFDGHECA-PVPIDGIVYMGA--GAGNRLQNLHRPRVVRGHCKVSKKKQVNVALNVGNCTG--FPGGDASTGW
S. NC---CRRWFLTFNGSECSSPTPIDAAMYVIG--PDN---SNPLRPRHIEGYCGGIGAGPVRVSLNVGNCPG--FGEADAFSGF
        * *::.**:. **  *  *:  .:            :      : * *         : . *            . :*:

H. NSVSRIIIEELPK-------
C. NSVSRLIIEELPK-------
B. NSVSRIIIEEIMMPEDSNSR
S. QSVSRIIIEEIPIAQ-----
L. ASATRIIVEEF---------
P. DSVSRMIIEEIPS-------
N. NAVSRIIVEEVPPQQT----
A. ISVSRIYVEEVPPPQA----
S. NAISRIIIEELPASPYS---
C. NSATRIYIEEVDQPQS----
S. ASASRIMIEEVAPPQP----
    : :*: :**.
```

Figure S3. Position of exon-intron boundaries in a selection of diverse metazoan *Cthrc1* genes. Schematic (top) and complete (bottom) alignments. The last AA of the first, second, third and fourth exons are marked in light green, pink and dark green respectively. The glycines of the CTHR domain are in orange (top). The alignment was done using MUSCLE.

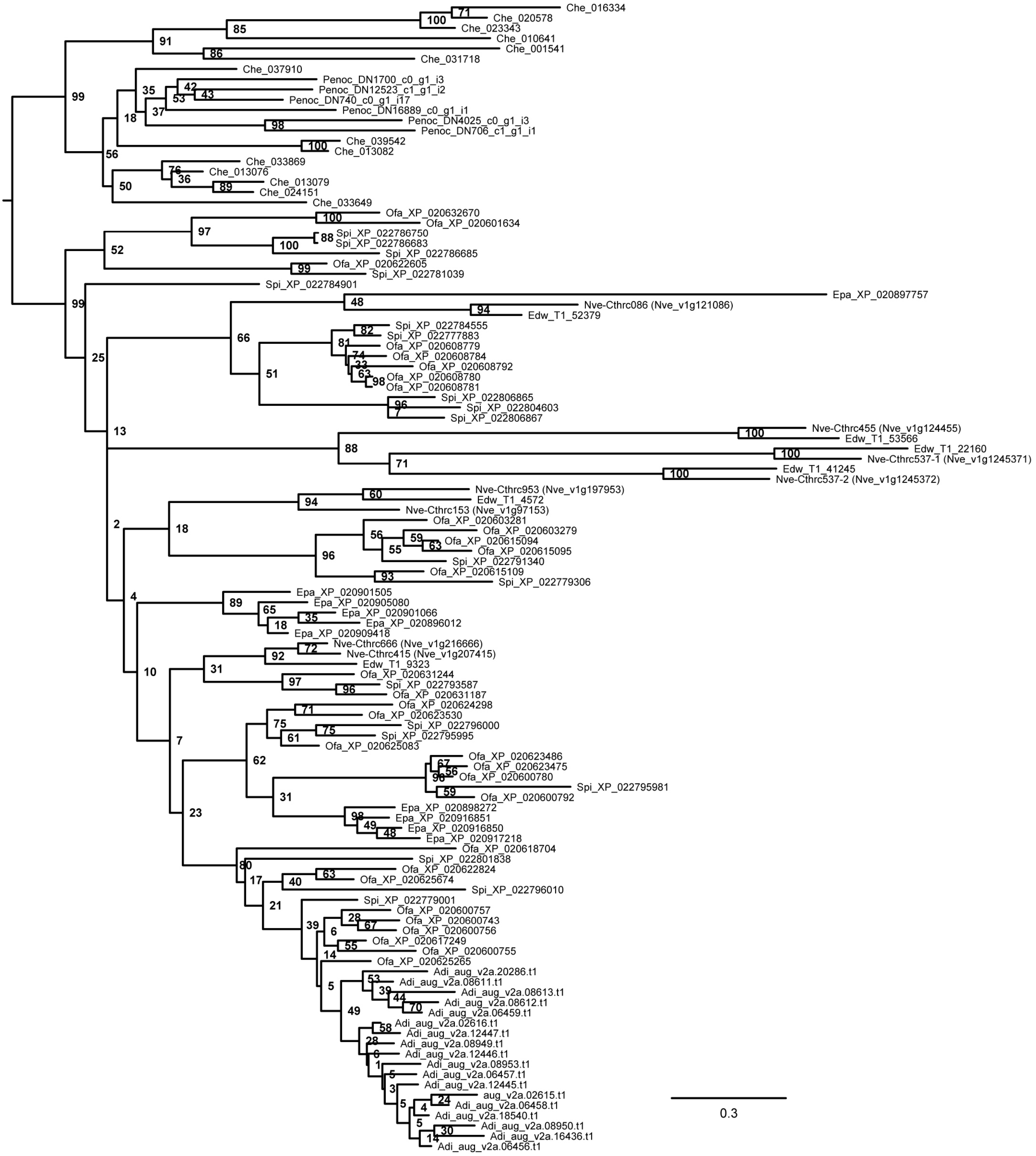

Figure S4. Maximum likelihood phylogeny of the Cthrc1 C-terminal domain for a selection of cnidarians (RAxML, model: WAG+G), as shown in Fig. 2B. ML Bootstrap values are indicated next to the nodes. The tree is arbitrarily rooted on the branch separating anthozoan and medusozoan *Cthrc1* genes. Scale bar: estimated number of substitution per site. Abbreviations - Adi: *Acropora digitifera*, Che: *Clytia hemisphaerica*, Epa: *Exaiptasia pallida*, Edw: *Edwardsiella lineata*, Nve: *Nematostella vectensis*, Ofa: *Orbicella faveolata*, Penoc: *Pelagia noctiluca*, Spi: *Stylophora pistillata*. The analysed alignment contains 141 positions for 112 sequences (703 positions for 112 sequences before trimming; see supplementary files).

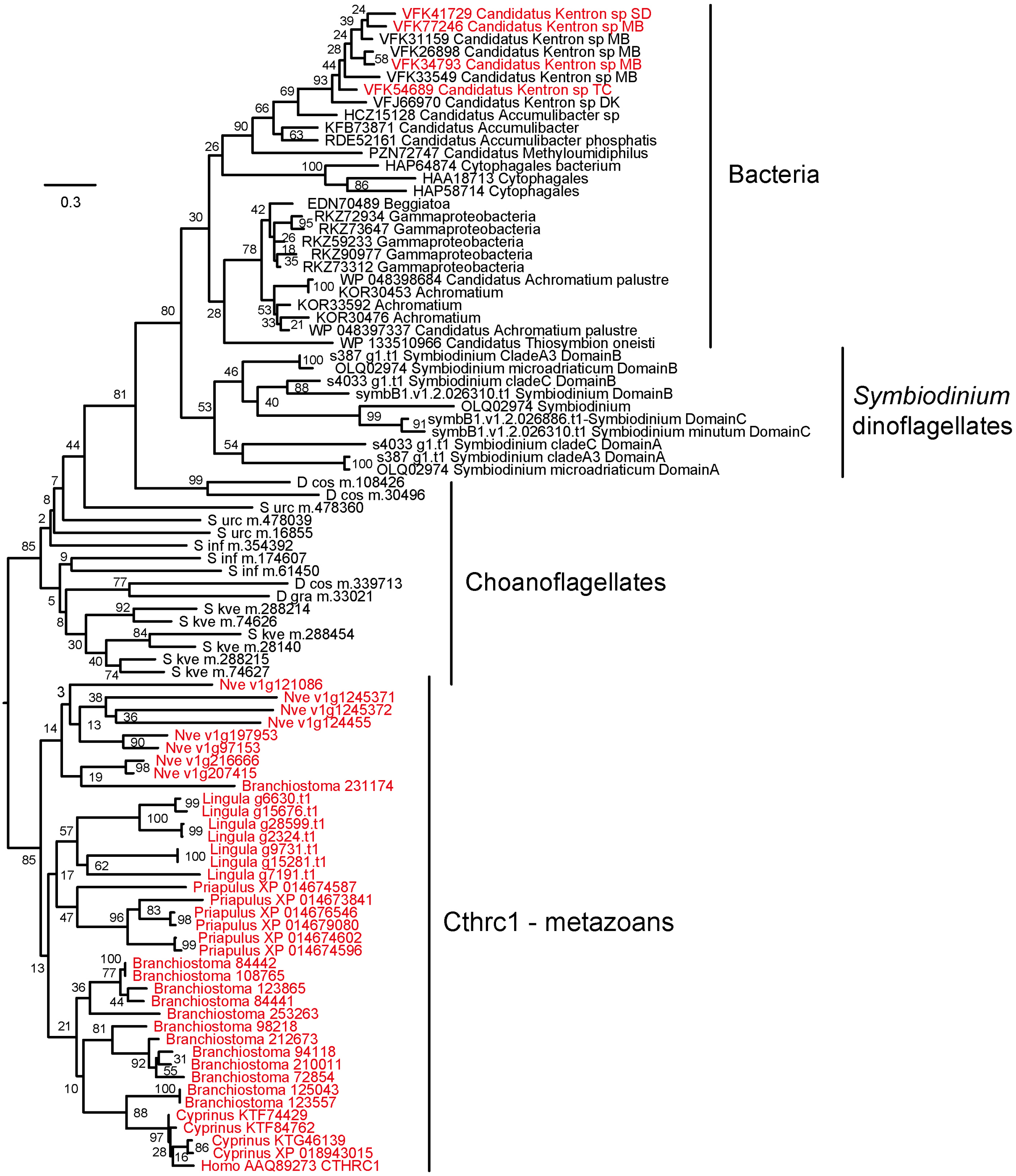

Figure S5. Maximum likelihood phylogeny of the Cthrc1 C-terminal domain including eukaryote and bacterial sequences (RAxML, model: LG+G). ML Bootstrap values are indicated next to the nodes. The tree is arbitrarily rooted on the branch separating metazoans and choanoflagellates *Cthrc1* C-terminal domain sequences. In red: *Cthrc1* C-terminal domain sequence linked to a CTHR domain. The retrieved *Symbiodinium* predicted proteins contain up to three Cthrc1 C-terminal domains called here A, B and C. Scale bar: estimated number of substitution per site. The analysed alignment contains 119 positions for 92 sequences (1762 positions for 101 sequences before trimming; see supplementary files).

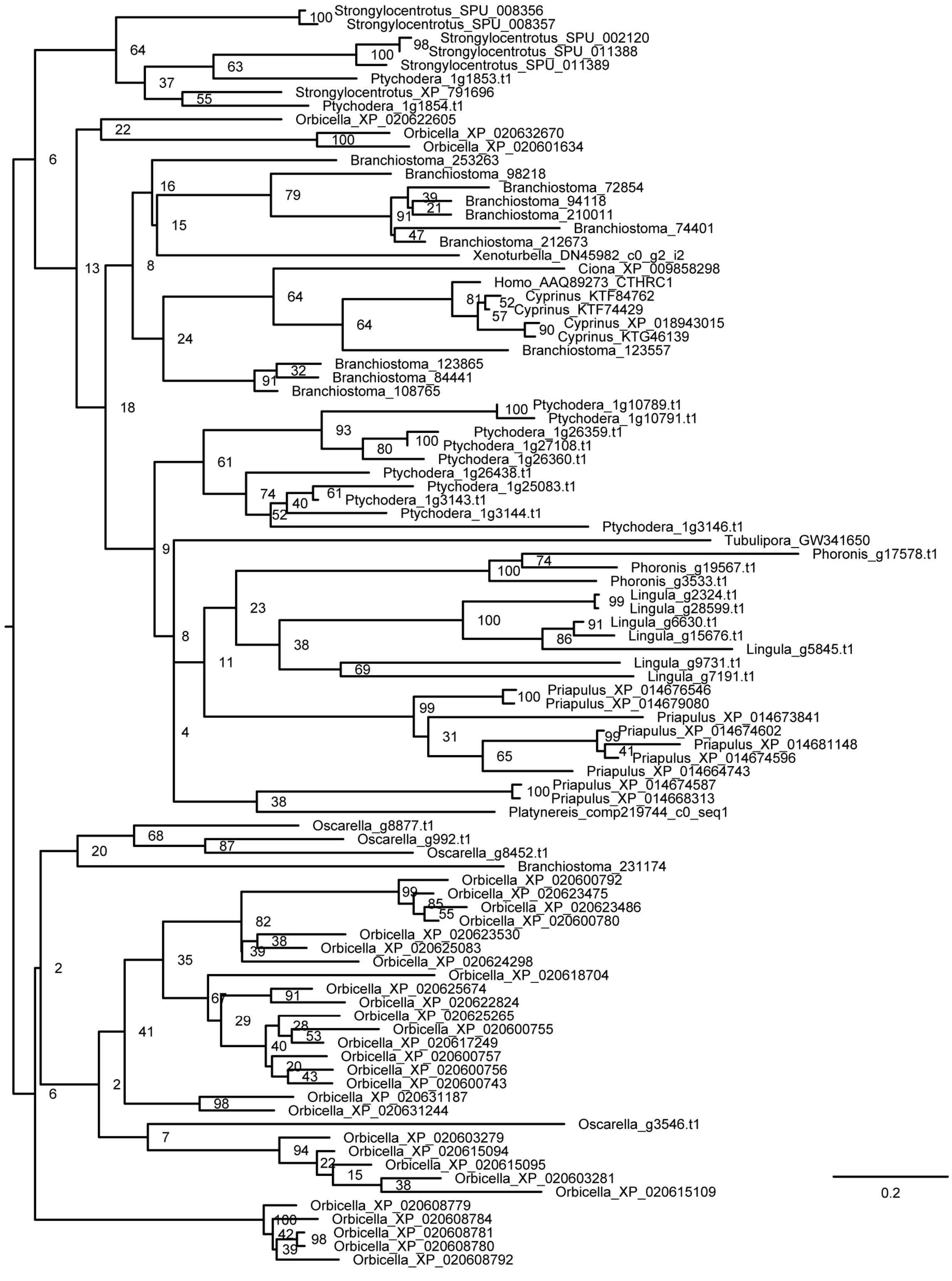

Figure S6. Maximum likelihood phylogeny of Cthrc1 C-terminal domain for a selection of metazoans (RAxML, model: WAG+G) as shown in Fig. 3B. ML Bootstrap values are indicated next to the nodes. The tree is arbitrarily rooted. Scale bar: estimated number of substitution per site. All identified *Cthrc1* genes for one species per clade were included (the one with the highest number in a given clade). The analysed alignment contains 135 positions for 93 sequences (1209 positions for 107 sequences before trimming; see supplementary files).

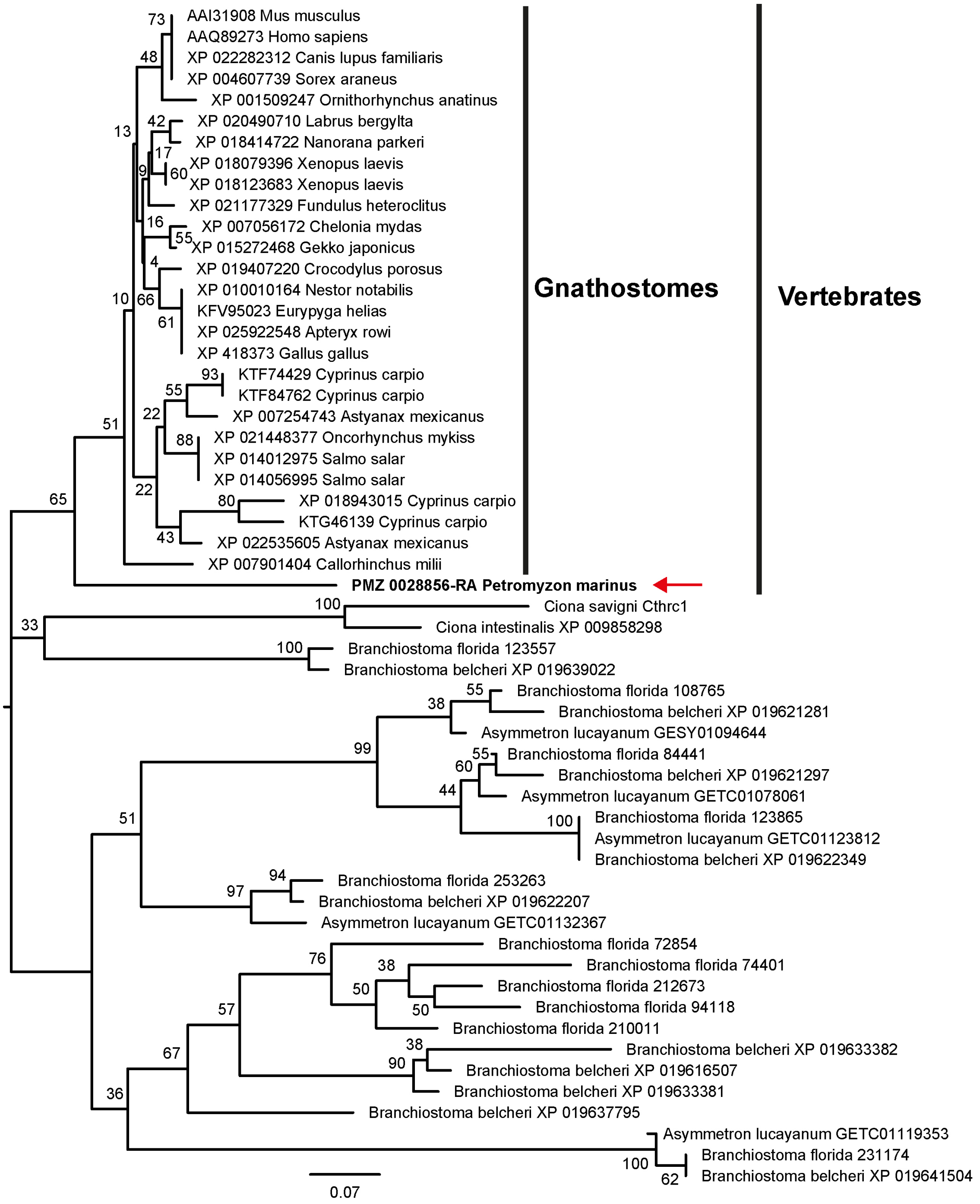

Figure S7. Phylogenetic analysis of chordate Cthrc1 including the partial sea lamprey sequence (red arrow) and a selection of chordates (BioNJ – Kimura model). Only the portion of the Cthrc1 C-terminal domain retrieved from the lamprey genome (~50 amino-acids) was included in the analysis. NJ Bootstrap values are indicated next to the nodes. The analysed alignment contains 47 positions for 56 sequences (314 positions for 59 sequences before trimming; see supplementary files). Scale bar: estimated number of substitution per site.

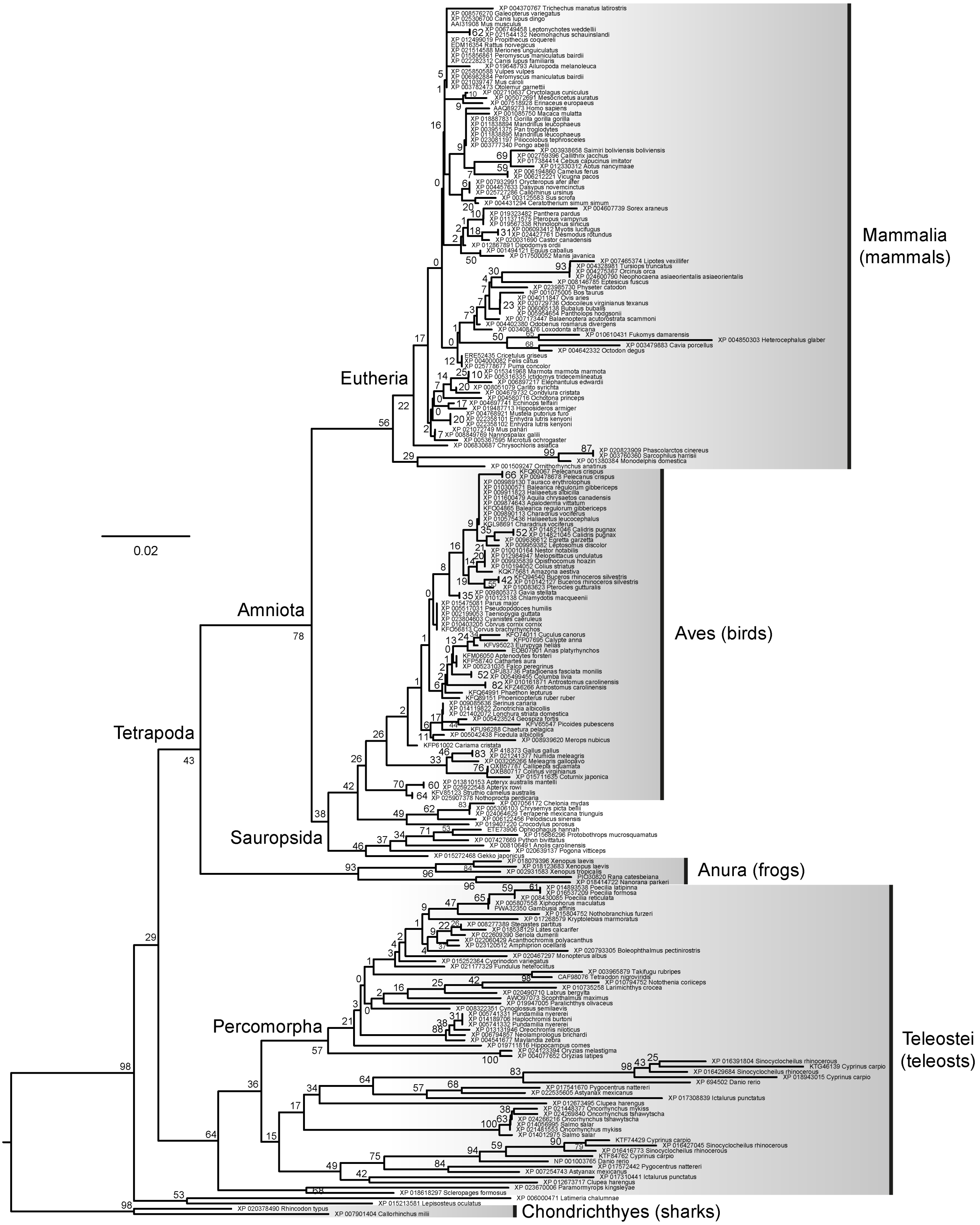

Figure S8. Phylogenetic analysis of vertebrate Cthrc1 (BioNJ – Kimura model) as shown in Fig. 4A and including all the Cthrc1 proteins retrieved from the NCBI database. Both the CTHR and the Cthrc1 C-terminal domains were included in the analysis. The tree is rooted using the shark's sequences. NJ Bootstrap values are indicated next to the nodes. Scale bar: estimated number of substitution per site. The analysed alignment contains 190 positions for 230 sequences (191 positions for 230 sequences before trimming; see supplementary files).

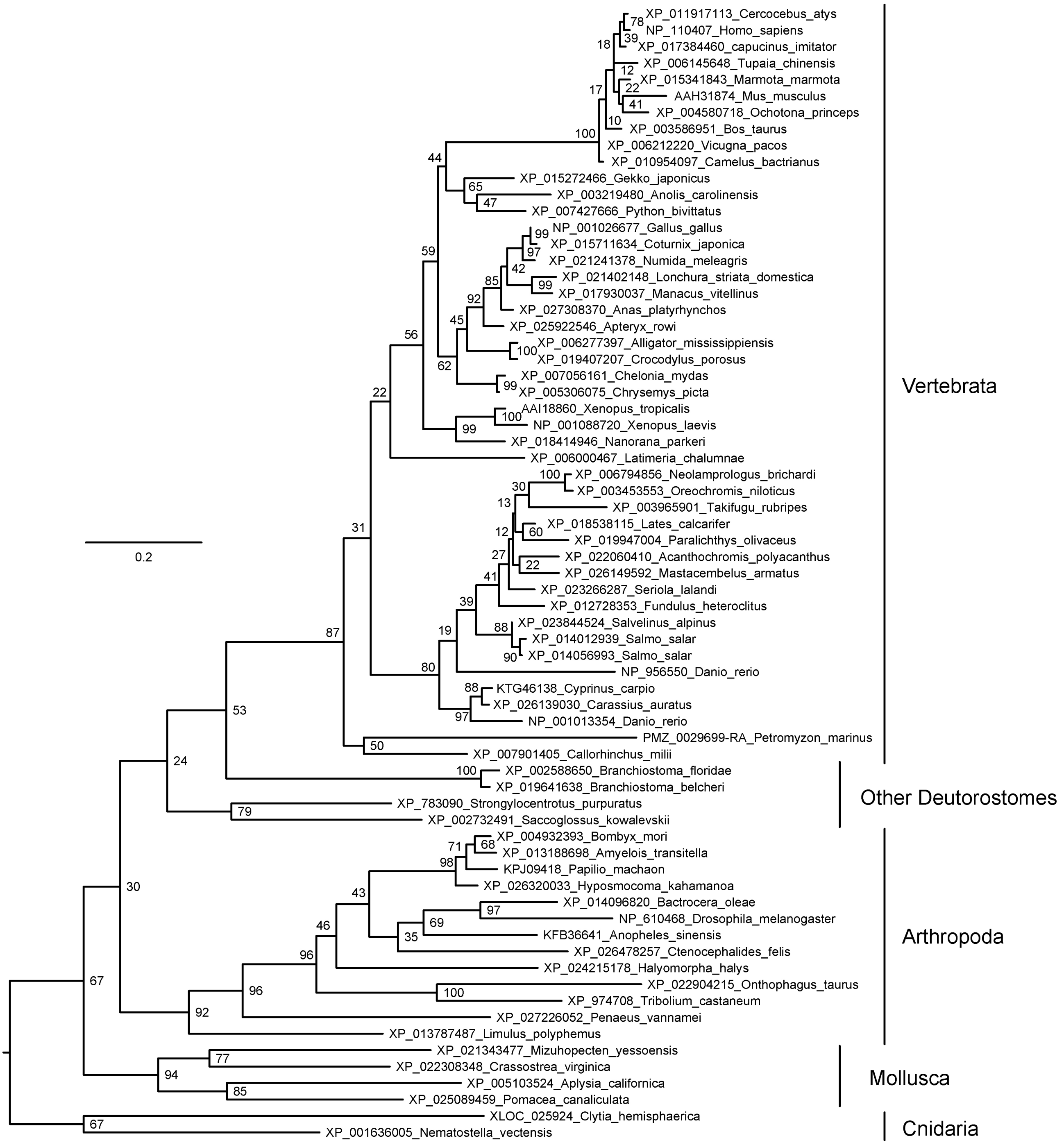

Figure S9. Maximum likelihood phylogeny of the mitochondrial folate carrier protein family Slc25a32 for a selection of metazoans (RAxML, model: JTT+G, BP) rooted with cnidarian sequences. ML Bootstrap values are indicated next to the nodes. The analysed alignment contains 306 positions for 69 sequences (392 positions for 70 sequences before trimming; see supplementary files).

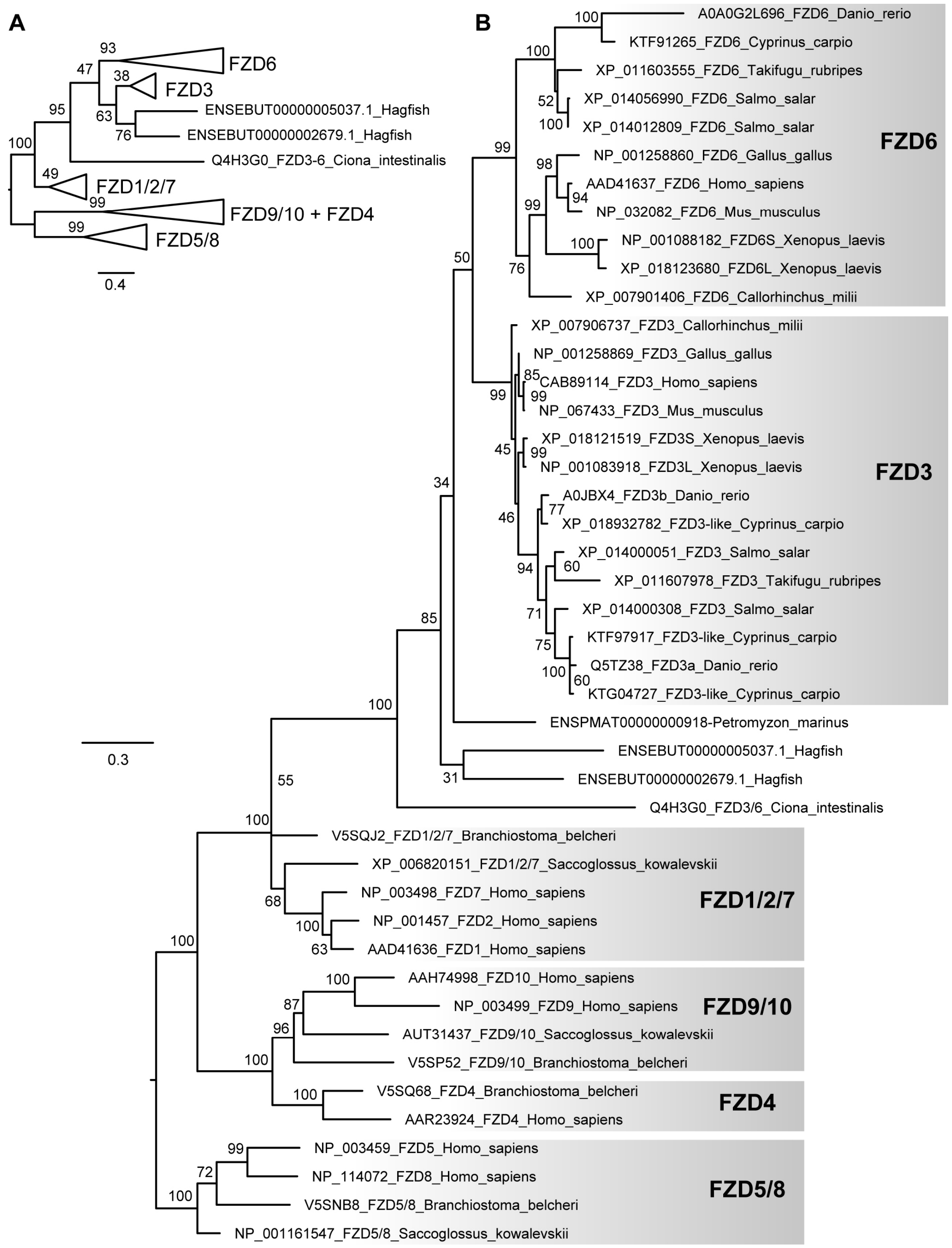

Figure S10. Phylogenetic analyses of the *Frizzled* gene family. ML phylogenetic analysis of Frizzled proteins (RAxML, model: JTT+G): (A) The CRD domain and (B) 7tm domain were analyzed separately (The sea lamprey *FZD3*/*FZD6*-like gene was not included in A as it does not contain a CRD domain). ML Bootstrap values are indicated next to the nodes. From these analyses, we can infer that the *FZD3* and *FZD6* gene families present in gnathostomes resulted from a duplication of an ancestral *FZD3/6* gene still present in the urochordate *Ciona intestinalis*. the *FZD3* or *FZD6*-like genes found in sea lamprey (*Petromyzon marinus*) and hagfish (*Eptatretus burgeri*) show conflicting and weakly supported phylogenetic positions – either sister to both *FZD3* and *FZD6* or sister to *FZD3* depending on the analyses. The analysed CRD and 7tm domain alignments contain respectively 179 and 363 positions for 43 and 44 sequences (1285 positions for 44 sequences before trimming and split between the CRD and 7tm domain alignments; see supplementary files).

A

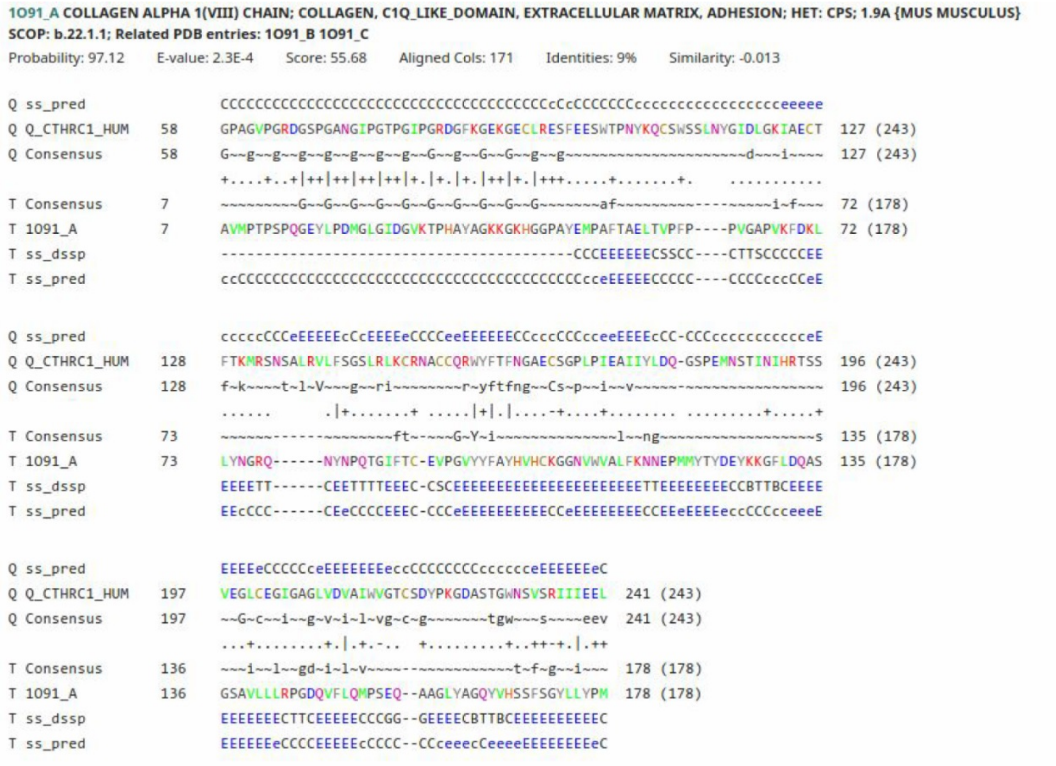

B

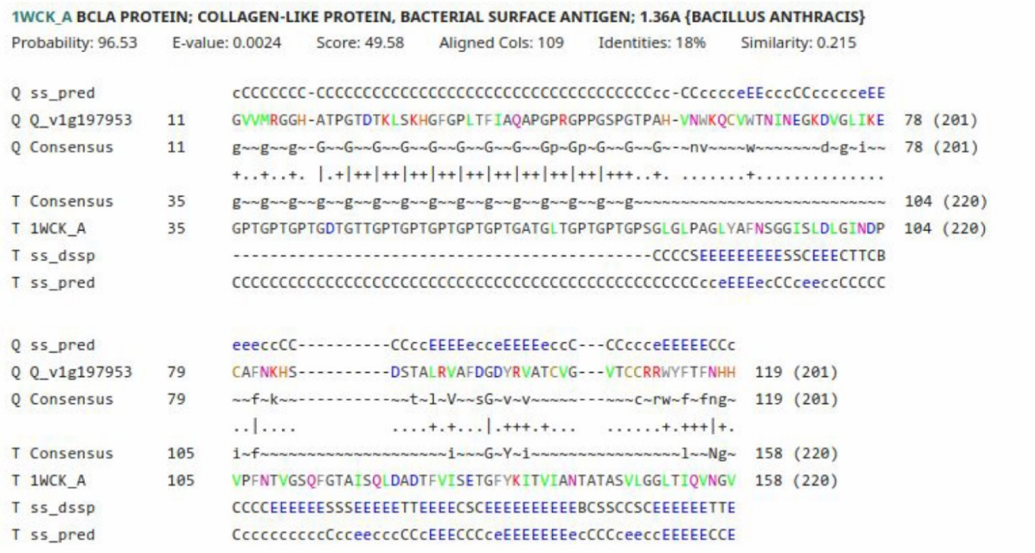

Figure S11. HHpred alignment scores of Cthrc1 and collagens. (A) Alignment of the CTHRC1 human protein against Collagen type VIII NC1 Domain, the top scoring eukaryotic homolog that was found by HHpred. (B) Alignment of the *Nematostella* Nve-Cthrc953 protein (v1g197953) against BclA, bacterial collagen-like protein, the top scoring homolog that was found by HHpred. The secondary structure is labeled with E for strands, C for coiled coils, G for 3-turn helices, T for hydrogen bonded turns, B for residue in isolated β-bridge and S for bends.

Table S1: Sequence Information about the *Nematostella* and *Clytia* Cthrc1 predicted proteins.

	Protein Name	Protein domains and their locations (Protein, aa)				Total length (aa)
		**Signal Peptide**	**position**	**Nb G-X-Y repeats**	**positions**	
Nematostella vectensis	Nve-Cthrc666	MKLSLVFVGVVQLSLILA	1-18	19	31-87	229
	Nve-Cthrc415	MLKFLLIFVGAVQLSLILA	1-19	20	32-91	232
	Nve-Cthrc086	MMKYLLIVSLVTALAMTAMT	1-20	30	52-141	285
	Nve-Cthrc153	MKVILSTCIFLLGLGYVAN	1-19	4	43-54	197
	Nve-Cthrc953	MSIILSMIILGVVMRGGHA	1-19	4	43-54	201
	Nve-Cthrc537-1	MQLLLLVFLCICGLSWG	1-17	12 & 19	39-74 & 86-142	258
	Nve-Cthrc537-2	MIYEIFFVISVTILCRNAVSVRS	1-23	12 & 20	42-77 & 91-150	297
	Nve-Cthrc455	MMNFSCFCLLWVVMLINIVSC	1-21	11 & 21	52-84 & 101-163	305
Clytia hemisphaerica	Che-Cthrc001541	MKIFHLFVYLFLLFNYHQC	1-19	10 & 32	54-83 & 102-197	322
	Che-Cthrc010641	MARIMMAVTVVLFLLFCNKFIVG	1-23	26	69-146	289
	Che-Cthrc013076	MKTVLTFFLLFVAVLGCIQA	1-20	7 & 8 & 19	32-51 & 54-137	280
	Che-Cthrc013079	MNKLICIPVLLTMMFVVVST	1-20	6 & 16	29-46 & 54-98	238
	Che-Cthrc013082	Missing N'	-	18 & 8 & 26	54-215	362
	Che-Cthrc016334	MKKIIFFIQILVVLGSTSS	1-19	30	54-140	271
	Che-Cthrc020578	MKKVILFIQILLVLGSTSS	1-19	30	53-139	272
	Che-Cthrc023343	MKKVILFIQILLVLGSTSST	1-20	31	54-143	273
	Che-Cthrc024151	MNKLICIPVLLAMMFVFVST	1-20	7 & 16	29-49 & 54-101	240
	Che-Cthrc031718	MAICSLFLAGLLISFIHG	1-18	17	48-98	238
	Che-Cthrc033649	MDFKIKLAIFLLSLSTISCHS	1-21	7	26-46	195
	Che-Cthrc033869	MTMKTVVFILLVTVLACTQA	1-20	36	32-138	281
	Che-Cthrc037910	MVKGFVAFRILLVFVVAVSCVCS	1-23	22	35-100	244
	Che-Cthrc039542	Missing N'	-	17 & 17 & 6	1-105 & 109-126	276

Table S2. Search for Cthrc1 C-terminal domain in non-metazoan genomes and transcriptomes.

Clade	Subclade	*Species*	Cthrc1 C-term domain	Blasted data	Source
Archaea		taxid: 2157	0	nr/nt	NCBI - 01/12/2019
Bacteria		taxd: 2	Yes**	nr/nt	NCBI - 01/12/2019
Archaeplastida	Amborellales	*Amborella trichopoda*	0	Genome	Ensembl Plants
Archaeplastida	Bryophyta	*Physcomitrella patens*	0	Genome	Ensembl Plants
Archaeplastida	Chlorophyta	*Chlamydomonas reinhardtii*	0	Genome	Ensembl Plants
Archaeplastida	Chlorophyta	*Ostreococcus lucimarinus*	0	Genome	Ensembl Plants
Archaeplastida	Chlorophyta	*Volvox carteri*	0	Genome	PlantGDB
Archaeplastida	eudicotyledons	*Actinidia chinensis*	0	Genome	Ensembl Plants
Archaeplastida	eudicotyledons	*Arabidopsis halleri*	0	Genome	Ensembl Plants
Archaeplastida	eudicotyledons	*Arabidopsis lyrata*	0	Genome	Ensembl Plants
Archaeplastida	eudicotyledons	*Arabidopsis thaliana*	0	Genome	Ensembl Plants
Archaeplastida	eudicotyledons	*Beta vulgaris*	0	Genome	Ensembl Plants
Archaeplastida	eudicotyledons	*Brassica napus*	0	Genome	Ensembl Plants
Archaeplastida	eudicotyledons	*Brassica oleracea*	0	Genome	Ensembl Plants
Archaeplastida	eudicotyledons	*Brassica rapa*	0	Genome	Ensembl Plants
Archaeplastida	eudicotyledons	*Capsicum annuum*	0	Genome	Ensembl Plants
Archaeplastida	eudicotyledons	*Coffea canephora*	0	Genome	Ensembl Plants
Archaeplastida	eudicotyledons	*Corchorus capsularis*	0	Genome	Ensembl Plants
Archaeplastida	eudicotyledons	*Cucumis sativus*	0	Genome	Ensembl Plants
Archaeplastida	eudicotyledons	*Cynara cardunculus*	0	Genome	Ensembl Plants
Archaeplastida	eudicotyledons	*Daucus carota*	0	Genome	Ensembl Plants
Archaeplastida	eudicotyledons	*Glycine max*	0	Genome	Ensembl Plants
Archaeplastida	eudicotyledons	*Gossypium raimondii*	0	Genome	Ensembl Plants
Archaeplastida	eudicotyledons	*Helianthus annuus*	0	Genome	Ensembl Plants
Archaeplastida	eudicotyledons	*Lupinus angustifolius*	0	Genome	Ensembl Plants
Archaeplastida	eudicotyledons	*Manihot esculenta*	0	Genome	Ensembl Plants
Archaeplastida	eudicotyledons	*Medicago truncatula*	0	Genome	Ensembl Plants
Archaeplastida	eudicotyledons	*Nicotiana attenuata*	0	Genome	Ensembl Plants
Archaeplastida	eudicotyledons	*Phaseolus vulgaris*	0	Genome	Ensembl Plants
Archaeplastida	eudicotyledons	*Populus trichocarpa*	0	Genome	Ensembl Plants
Archaeplastida	eudicotyledons	*Prunus persica*	0	Genome	Ensembl Plants
Archaeplastida	eudicotyledons	*Solanum lycopersicum*	0	Genome	Ensembl Plants
Archaeplastida	eudicotyledons	*Solanum tuberosum*	0	Genome	Ensembl Plants
Archaeplastida	eudicotyledons	*Theobroma cacao*	0	Genome	Ensembl Plants
Archaeplastida	eudicotyledons	*Trifolium pratense*	0	Genome	Ensembl Plants
Archaeplastida	eudicotyledons	*Vigna angularis*	0	Genome	Ensembl Plants
Archaeplastida	eudicotyledons	*Vigna radiata*	0	Genome	Ensembl Plants
Archaeplastida	eudicotyledons	*Vitis vinifera*	0	Genome	Ensembl Plants
Archaeplastida	Liliopsida	*Aegilops tauschii*	0	Genome	Ensembl Plants
Archaeplastida	Liliopsida	*Brachypodium distachyon*	0	Genome	Ensembl Plants
Archaeplastida	Liliopsida	*Dioscorea rotundata*	0	Genome	Ensembl Plants
Archaeplastida	Liliopsida	*Eragrostis tef*	0	Genome	Ensembl Plants
Archaeplastida	Liliopsida	*Hordeum vulgare*	0	Genome	Ensembl Plants
Archaeplastida	Liliopsida	*Leersia perrieri*	0	Genome	Ensembl Plants
Archaeplastida	Liliopsida	*Musa acuminata*	0	Genome	Ensembl Plants
Archaeplastida	Liliopsida	*Oryza barthii*	0	Genome	Ensembl Plants
Archaeplastida	Liliopsida	*Oryza brachyantha*	0	Genome	Ensembl Plants
Archaeplastida	Liliopsida	*Oryza glaberrima*	0	Genome	Ensembl Plants
Archaeplastida	Liliopsida	*Oryza glumipatula*	0	Genome	Ensembl Plants
Archaeplastida	Liliopsida	*Oryza longistaminata*	0	Genome	Ensembl Plants
Archaeplastida	Liliopsida	*Oryza meridionalis*	0	Genome	Ensembl Plants
Archaeplastida	Liliopsida	*Oryza nivara*	0	Genome	Ensembl Plants
Archaeplastida	Liliopsida	*Oryza punctata*	0	Genome	Ensembl Plants
Archaeplastida	Liliopsida	*Oryza rufipogon*	0	Genome	Ensembl Plants
Archaeplastida	Liliopsida	*Oryza sativa Indica Group*	0	Genome	Ensembl Plants

Archaeplastida	Liliopsida	*Oryza sativa Japonica Group*	0	Genome	Ensembl Plants
Archaeplastida	Liliopsida	*Panicum hallii FIL2*	0	Genome	Ensembl Plants
Archaeplastida	Liliopsida	*Setaria italica*	0	Genome	Ensembl Plants
Archaeplastida	Liliopsida	*Sorghum bicolor*	0	Genome	Ensembl Plants
Archaeplastida	Liliopsida	*Triticum aestivum*	0	Genome	Ensembl Plants
Archaeplastida	Liliopsida	*Triticum dicoccoides*	0	Genome	Ensembl Plants
Archaeplastida	Liliopsida	*Triticum turgidum*	0	Genome	Ensembl Plants
Archaeplastida	Liliopsida	*Triticum urartu*	0	Genome	Ensembl Plants
Archaeplastida	Liliopsida	*Zea mays*	0	Genome	Ensembl Plants
Archaeplastida	Lycopodiophyta	*Selaginella moellendorffii*	0	Genome	Ensembl Plants
Archaeplastida	Marchantiophyta	*Marchantia polymorpha*	0	Genome	Ensembl Plants
Archaeplastida	Rhodophyta	*Chondrus crispus*	0	Genome	Ensembl Plants
Archaeplastida	Rhodophyta	*Cyanidioschyzon merolae*	0	Genome	Ensembl Plants
Archaeplastida	Rhodophyta	*Galdieria sulphuraria*	0	Genome	Ensembl Plants
Alveolata	Apicomplexa	*Babesia bigemina*	0	Genome	Ensembl Protists
Alveolata	Apicomplexa	*Babesia bovis*	0	Genome	Ensembl Protists
Alveolata	Apicomplexa	*Babesia ovata*	0	Genome	Ensembl Protists
Alveolata	Apicomplexa	*Besnoitia besnoiti*	0	Genome	Ensembl Protists
Alveolata	Apicomplexa	*Cryptosporidium andersoni*	0	Genome	Ensembl Protists
Alveolata	Apicomplexa	*Cryptosporidium meleagridis*	0	Genome	Ensembl Protists
Alveolata	Apicomplexa	*Cryptosporidium muris*	0	Genome	Ensembl Protists
Alveolata	Apicomplexa	*Cryptosporidium parvum*	0	Genome	Ensembl Protists
Alveolata	Apicomplexa	*Cryptosporidium ubiquitum*	0	Genome	Ensembl Protists
Alveolata	Apicomplexa	*Cyclospora cayetanensis*	0	Genome	Ensembl Protists
Alveolata	Apicomplexa	*Cystoisospora suis*	0	Genome	Ensembl Protists
Alveolata	Apicomplexa	*Eimeria acervulina*	0	Genome	Ensembl Protists
Alveolata	Apicomplexa	*Eimeria brunetti*	0	Genome	Ensembl Protists
Alveolata	Apicomplexa	*Eimeria maxima*	0	Genome	Ensembl Protists
Alveolata	Apicomplexa	*Eimeria mitis*	0	Genome	Ensembl Protists
Alveolata	Apicomplexa	*Eimeria praecox*	0	Genome	Ensembl Protists
Alveolata	Apicomplexa	*Eimeria tenella*	0	Genome	Ensembl Protists
Alveolata	Apicomplexa	*Gregarina niphandrodes*	0	Genome	Ensembl Protists
Alveolata	Apicomplexa	*Hammondia hammondi*	0	Genome	Ensembl Protists
Alveolata	Apicomplexa	*Plasmodium berghei*	0	Genome	Ensembl Protists
Alveolata	Apicomplexa	*Plasmodium chabaudi*	0	Genome	Ensembl Protists
Alveolata	Apicomplexa	*Plasmodium coatneyi*	0	Genome	Ensembl Protists
Alveolata	Apicomplexa	*Plasmodium cynomolgi*	0	Genome	Ensembl Protists
Alveolata	Apicomplexa	*Plasmodium falciparum*	0	Genome	Ensembl Protists
Alveolata	Apicomplexa	*Plasmodium fragile*	0	Genome	Ensembl Protists
Alveolata	Apicomplexa	*Plasmodium gaboni*	0	Genome	Ensembl Protists
Alveolata	Apicomplexa	*Plasmodium gallinaceum*	0	Genome	Ensembl Protists
Alveolata	Apicomplexa	*Plasmodium gonderi*	0	Genome	Ensembl Protists
Alveolata	Apicomplexa	*Plasmodium knowlesi*	0	Genome	Ensembl Protists
Alveolata	Apicomplexa	*Plasmodium malariae*	0	Genome	Ensembl Protists
Alveolata	Apicomplexa	*Plasmodium ovale*	0	Genome	Ensembl Protists
Alveolata	Apicomplexa	*Plasmodium reichenow*	0	Genome	Ensembl Protists
Alveolata	Apicomplexa	*Plasmodium relictum*	0	Genome	Ensembl Protists
Alveolata	Apicomplexa	*Plasmodium vinckei*	0	Genome	Ensembl Protists
Alveolata	Apicomplexa	*Plasmodium vivax*	0	Genome	Ensembl Protists
Alveolata	Apicomplexa	*Plasmodium yoelii*	0	Genome	Ensembl Protists
Alveolata	Apicomplexa	*Theileria orientalis*	0	Genome	Ensembl Protists
Alveolata	Apicomplexa	*Theileria parva*	0	Genome	Ensembl Protists
Alveolata	Apicomplexa	*Toxoplasma gondii*	0	Genome	Ensembl Protists
Alveolata	Cliophora	*Ichthyophthirius multifillis*	0	Genome	Ensembl Protists
Alveolata	Cliophora	*Oxytricha trifallax*	0	Genome	Ensembl Protists
Alveolata	Cliophora	*Paramecium tetraurelia*	0	Genome	Ensembl Protists
Alveolata	Cliophora	*Pseudocohnilembus persalinus*	0	Genome	Ensembl Protists
Alveolata	Cliophora	*Stentor coeruleus*	0	Genome	Ensembl Protists
Alveolata	Cliophora	*Stylonychia lemnae*	0	Genome	Ensembl Protists

Alveolata	Cliophora	*Tetrahymena thermophila*	0	Genome	Ensembl Protists
Alveolata	Dinophyta	*Perkinsus marinus*	0	Genome	Ensembl Protists
Alveolata	Dinophyta	*Polarella_glacialis*	0	Genome	Stephens et al. 2019
Alveolata	Dinophyta	*Symbiodinium microadriaticum*	1*	Genome	Ensembl Protists
Alveolata	Dinophyta	*Symbiodinium minutum*	2*	Genome	marinegenomics.oist.jp
Alveolata	Dinophyta	*Symbiodinium muscatinei*	1*	Transcriptome	NCBI TSA
Alveolata	Dinophyta	*Symbiodinium sp. A1*	1*	Transcriptome	NCBI TSA
Alveolata	Dinophyta	*Symbiodinium sp. A2*	1*	Transcriptome	NCBI TSA
Alveolata	Dinophyta	*Symbiodinium sp. A3*	1*	Genome	marinegenomics.oist.jp
Alveolata	Dinophyta	*Symbiodinium sp. clade C*	1*	Genome	marinegenomics.oist.jp
Alveolata	Dinophyta	*Symbiodinium sp. clade D*	1*	Transcriptome	NCBI TSA
Amoebozoa	Discosea	*Acanthamoeba castellanii*	0	Genome	Ensembl Protists
Amoebozoa	Entamoeba	*Acanthamoeba castellanii*	0	Genome	Ensembl Protists
Amoebozoa	Entamoeba	*Entamoeba dispar SAW760*	0	Genome	Ensembl Protists
Amoebozoa	Entamoeba	*Entamoeba histolytica*	0	Genome	Ensembl Protists
Amoebozoa	Entamoeba	*Entamoeba invadens*	0	Genome	Ensembl Protists
Amoebozoa	Entamoeba	*Entamoeba nuttalli*	0	Genome	Ensembl Protists
Amoebozoa	Mycetozoa	*Acanthamoeba castellanii*	0	Genome	Ensembl Protists
Amoebozoa	Mycetozoa	*Cavenderia fasciculata*	0	Genome	Ensembl Protists
Amoebozoa	Mycetozoa	*Dictyostelium discoideum*	0	Genome	Ensembl Protists
Amoebozoa	Mycetozoa	*Dictyostelium discoideum*	0	Genome	Ensembl Protists
Amoebozoa	Mycetozoa	*Dictyostelium purpureum*	0	Genome	Ensembl Protists
Amoebozoa	Mycetozoa	*Dictyostelium purpureum*	0	Genome	Ensembl Protists
Amoebozoa	Mycetozoa	*Physarum polycephalum*	0	Genome	Ensembl Protists
Amoebozoa	Mycetozoa	*Planoprotostelium fungivorum*	0	Genome	Ensembl Protists
Amoebozoa	Mycetozoa	*Tieghemostelium lacteum*	0	Genome	Ensembl Protists
Apusozoa	Thecomonadea	*Thecamonas trahens*	0	Genome	Ensembl Protists
Cryptophyta	Cryptomonadales	*Cryptomonas paramecium*	0	Genome	Ensembl Protists
Cryptophyta	Pyrenomonadales	*Chroomonas mesostigmatica*	0	Genome	Ensembl Protists
Cryptophyta	Pyrenomonadales	*Guillardia theta*	0	Genome	Ensembl Protists
Excavata	Euglenozoa	*Angomonas deanei*	0	Genome	Ensembl Protists
Excavata	Euglenozoa	*Leishmania donovani*	0	Genome	Ensembl Protists
Excavata	Euglenozoa	*Leishmania infantum*	0	Genome	Ensembl Protists
Excavata	Euglenozoa	*Leishmania major*	0	Genome	Ensembl Protists
Excavata	Euglenozoa	*Leishmania panamensis*	0	Genome	Ensembl Protists
Excavata	Euglenozoa	*Leptomonas pyrrhocoris*	0	Genome	Ensembl Protists
Excavata	Euglenozoa	*Leptomonas seymouri*	0	Genome	Ensembl Protists
Excavata	Euglenozoa	*Perkinsela sp.*	0	Genome	Ensembl Protists
Excavata	Euglenozoa	*Phytomonas sp.*	0	Genome	Ensembl Protists
Excavata	Euglenozoa	*Strigomonas culicis*	0	Genome	Ensembl Protists
Excavata	Euglenozoa	*Trypanosoma brucei*	0	Genome	Ensembl Protists
Excavata	Euglenozoa	*Trypanosoma conorhini*	0	Genome	Ensembl Protists
Excavata	Euglenozoa	*Trypanosoma cruzi*	0	Genome	Ensembl Protists
Excavata	Euglenozoa	*Trypanosoma equiperdum*	0	Genome	Ensembl Protists
Excavata	Euglenozoa	*Trypanosoma rangeli*	0	Genome	Ensembl Protists
Excavata	Euglenozoa	*Trypanosoma theileri*	0	Genome	Ensembl Protists
Excavata	Fornicata	*Giardia intestinalis*	0	Genome	Ensembl Protists
Excavata	Fornicata	*Kipferlia bialata*	0	Genome	Ensembl Protists
Excavata	Fornicata	*Spironucleus salmonicida*	0	Genome	Ensembl Protists
Excavata	Parabasalia	*Tritrichomonas foetus*	0	Genome	Ensembl Protists
Excavata	Percolozoa	*Naegleria gruberi str. NEG-M*	0	Genome	Ensembl Protists
Haptophyta	Coccolithophyceae	*Emiliania huxleyi*	0	Genome	Ensembl Protists
Rhizaria	Cercozoa	*Bigelowiella natans*	0	Genome	Ensembl Protists
Rhizaria	Cercozoa	*Plasmodiophora brassicae*	0	Genome	Ensembl Protists
Rhizaria	Foraminifera	*Reticulomyxa filosa*	0	Genome	Ensembl Protists
Stramenopiles	Bacillariophyta	*Fragilariopsis cylindrus*	0	Genome	Ensembl Protists
Stramenopiles	Bacillariophyta	*Phaeodactylum tricornutum*	0	Genome	Ensembl Protists
Stramenopiles	Bacillariophyta	*Thalassiosira oceanica*	0	Genome	Ensembl Protists
Stramenopiles	Bacillariophyta	*Thalassiosira pseudonana*	0	Genome	Ensembl Protists

Stramenopiles	Blastocystae	*Blastocystis hominis*	0	Genome	Ensembl Protists
Stramenopiles	Eustigmatophyceae	*Nannochloropsis gaditana*	0	Genome	Ensembl Protists
Stramenopiles	Labyrinthulomycetes	*Hondaea fermentalgiana*	0	Genome	Ensembl Protists
Stramenopiles	Pelagophyceae	*Aureococcus anophagefferens*	0	Genome	Ensembl Protists
Stramenopiles	Phaeophyceae	*Ectocarpus siliculosus*	0	Genome	Ensembl Protists
Stramenopiles	Oomycota	*Achlya hypogyna*	0	Genome	Ensembl Protists
Stramenopiles	Oomycota	*Albugo laibachii*	0	Genome	Ensembl Protists
Stramenopiles	Oomycota	*Aphanomyces astaci*	0	Genome	Ensembl Protists
Stramenopiles	Oomycota	*Aphanomyces invadans*	0	Genome	Ensembl Protists
Stramenopiles	Oomycota	*Hyaloperonospora arabidopsidis*	0	Genome	Ensembl Protists
Stramenopiles	Oomycota	*Nothophytophthora sp. Chile5*	0	Genome	Ensembl Protists
Stramenopiles	Oomycota	*Peronospora effusa*	0	Genome	Ensembl Protists
Stramenopiles	Oomycota	*Phytophthora cactorum*	0	Genome	Ensembl Protists
Stramenopiles	Oomycota	*Phytophthora infestans*	0	Genome	Ensembl Protists
Stramenopiles	Oomycota	*Phytophthora kernoviae*	0	Genome	Ensembl Protists
Stramenopiles	Oomycota	*Phytophthora lateralis*	0	Genome	Ensembl Protists
Stramenopiles	Oomycota	*Phytophthora megakarya*	0	Genome	Ensembl Protists
Stramenopiles	Oomycota	*Phytophthora nicotianae*	0	Genome	Ensembl Protists
Stramenopiles	Oomycota	*Phytophthora palmivora*	0	Genome	Ensembl Protists
Stramenopiles	Oomycota	*Phytophthora parasitica*	0	Genome	Ensembl Protists
Stramenopiles	Oomycota	*Phytophthora ramorum*	0	Genome	Ensembl Protists
Stramenopiles	Oomycota	*Phytophthora sojae*	0	Genome	Ensembl Protists
Stramenopiles	Oomycota	*Plasmopara halstedii*	0	Genome	Ensembl Protists
Stramenopiles	Oomycota	*Pythium aphanidermatum*	0	Genome	Ensembl Protists
Stramenopiles	Oomycota	*Pythium arrhenomanes*	0	Genome	Ensembl Protists
Stramenopiles	Oomycota	*Pythium irregulare*	0	Genome	Ensembl Protists
Stramenopiles	Oomycota	*Pythium iwayamai*	0	Genome	Ensembl Protists
Stramenopiles	Oomycota	*Saprolegnia diclina*	0	Genome	Ensembl Protists
Stramenopiles	Oomycota	*Saprolegnia parasitica*	0	Genome	Ensembl Protists
Stramenopiles	Oomycota	*Thraustotheca clavata*	0	Genome	Ensembl Protists
Opisthokonta	Ascomyocota	*Acidomyces richmondensis (GCA_001572075)*	0	Genome	Ensembl Fungi
Opisthokonta	Ascomyocota	*Diplodia seriata (GCA_001006355)*	0	Genome	Ensembl Fungi
Opisthokonta	Ascomyocota	*Baudoinia panamericana UAMH 10762 (GCA_000338955)*	0	Genome	Ensembl Fungi
Opisthokonta	Ascomyocota	*Dothistroma septosporum*	0	Genome	Ensembl Fungi
Opisthokonta	Ascomyocota	*Rachicladosporium antarcticum (GCA_002077065)*	0	Genome	Ensembl Fungi
Opisthokonta	Ascomyocota	*Ramularia collo-cygni (GCA_900074925)*	0	Genome	Ensembl Fungi
Opisthokonta	Ascomyocota	*Capronia coronata CBS 617.96 (GCA_000585585)*	0	Genome	Ensembl Fungi
Opisthokonta	Ascomyocota	*Exophiala mesophila str. CBS 40295 (GCA_000836275)*	0	Genome	Ensembl Fungi
Opisthokonta	Ascomyocota	*Fonsecaea erecta (GCA_001651985)*	0	Genome	Ensembl Fungi
Opisthokonta	Ascomyocota	*Phialophora americana str. CBS 27337 (GCA_000835435)*	0	Genome	Ensembl Fungi
Opisthokonta	Ascomyocota	*Rhinocladiella mackenziei CBS 650.93 (GCA_000835555)*	0	Genome	Ensembl Fungi
Opisthokonta	Ascomyocota	*Aureobasidium subglaciale EXF-2481 (GCA_000721755)*	0	Genome	Ensembl Fungi
Opisthokonta	Ascomyocota	*Hortaea werneckii EXF-2000 (GCA_002127715)*	0	Genome	Ensembl Fungi
Opisthokonta	Ascomyocota	*Blumeria graminis f. sp. tritici 96224 (GCA_000418435)*	0	Genome	Ensembl Fungi
Opisthokonta	Ascomyocota	*Erysiphe necator (GCA_000798715)*	0	Genome	Ensembl Fungi
Opisthokonta	Ascomyocota	*Aspergillus aculeatinus CBS 121060 (GCA_003184765)*	0	Genome	Ensembl Fungi
Opisthokonta	Ascomyocota	*Aspergillus flavus AF70 (GCA_000952835)*	0	Genome	Ensembl Fungi
Opisthokonta	Ascomyocota	*Byssochlamys spectabilis No. 5*	0	Genome	Ensembl Fungi
Opisthokonta	Ascomyocota	*Penicillium freii (GCA_001513925)*	0	Genome	Ensembl Fungi
Opisthokonta	Ascomyocota	*Colletotrichum chlorophyti str. NTL11 (GCA_001937105)*	0	Genome	Ensembl Fungi
Opisthokonta	Ascomyocota	*Verticillium dahliae (GCA_002893025)*	0	Genome	Ensembl Fungi
Opisthokonta	Ascomyocota	*Glarea lozoyensis ATCC 20868 str. 1224 (GCA_000409485)*	0	Genome	Ensembl Fungi
Opisthokonta	Ascomyocota	*Pezoloma ericae str. UAMH 7357 (GCA_002865625)*	0	Genome	Ensembl Fungi
Opisthokonta	Ascomyocota	*Rutstroemia sp. NJR-2017a BBW (GCA_002946425)*	0	Genome	Ensembl Fungi
Opisthokonta	Ascomyocota	*Acremonium chrysogenum ATCC 11550 (GCA_000769265)*	0	Genome	Ensembl Fungi
Opisthokonta	Ascomyocota	*Trichoderma reesei*	0	Genome	Ensembl Fungi
Opisthokonta	Ascomyocota	*Magnaporthe poae*	0	Genome	Ensembl Fungi
Opisthokonta	Ascomyocota	*Ceratocystis platani str. CFO (GCA_000978885)*	0	Genome	Ensembl Fungi
Opisthokonta	Ascomyocota	*Amorphotheca resinae ATCC 22711 (GCA_003019875)*	0	Genome	Ensembl Fungi

Opisthokonta	Ascomyocota	*Oidiodendron maius Zn (GCA_000827325)*	0	Genome	Ensembl Fungi
Opisthokonta	Ascomyocota	*Ascosphaera apis ARSEF 7405 (GCA_001636715)*	0	Genome	Ensembl Fungi
Opisthokonta	Ascomyocota	*Trichophyton mentagrophytes str. TIMM 2789 (GCA_003118255)*	0	Genome	Ensembl Fungi
Opisthokonta	Ascomyocota	*Grosmannia clavigera kw1407 (GCA_000143105)*	0	Genome	Ensembl Fungi
Opisthokonta	Ascomyocota	*Arthrobotrys oligospora ATCC 24927 (GCA_000225545)*	0	Genome	Ensembl Fungi
Opisthokonta	Ascomyocota	*Tuber borchii str. Tbo3840 (GCA_003070745)*	0	Genome	Ensembl Fungi
Opisthokonta	Ascomyocota	*Alternaria alternata str. SRC1lrK2f (GCA_001642055)*	0	Genome	Ensembl Fungi
Opisthokonta	Ascomyocota	*Clohesyomyces aquaticus str. CBS 115471 (GCA_002105025)*	0	Genome	Ensembl Fungi
Opisthokonta	Ascomyocota	*Pseudogymnoascus destructans (GCA_001641265)*	0	Genome	Ensembl Fungi
Opisthokonta	Ascomyocota	*Babjeviella inositovora NRRL Y-12698 (GCA_001661335)*	0	Genome	Ensembl Fungi
Opisthokonta	Ascomyocota	*Candida albicans L26 (GCA_000775455)*	0	Genome	Ensembl Fungi
Opisthokonta	Ascomyocota	*Saccharomyces cerevisiae (GCA_001634645)*	0	Genome	Ensembl Fungi
Opisthokonta	Ascomyocota	*Schizosaccharomyces pombe*	0	Genome	Ensembl Fungi
Opisthokonta	Ascomyocota	*Botrytis cinerea B05.10*	0	Genome	Ensembl Fungi
Opisthokonta	Ascomyocota	*Sclerotinia borealis F-4128 (GCA_000503235)*	0	Genome	Ensembl Fungi
Opisthokonta	Ascomyocota	*Chaetomium thermophilum DSM 1495 (GCA_000221225)*	0	Genome	Ensembl Fungi
Opisthokonta	Ascomyocota	*Coniella lustricola str. B22-T-1 (GCA_003019895)*	0	Genome	Ensembl Fungi
Opisthokonta	Ascomyocota	*Neolecta irregularis DAH-3 (GCA_001929475)*	0	Genome	Ensembl Fungi
Opisthokonta	Ascomyocota	*Verruconis gallopava str. CBS 43764 (GCA_000836295)*	0	Genome	Ensembl Fungi
Opisthokonta	Ascomyocota	*Endocarpon pusillum Z07020 (GCA_000464535)*	0	Genome	Ensembl Fungi
Opisthokonta	Ascomyocota	*Eutypa lata UCREL1 (GCA_000349385)*	0	Genome	Ensembl Fungi
Opisthokonta	Ascomyocota	*Elsinoe australis str. NL1 (GCA_003013795)*	0	Genome	Ensembl Fungi
Opisthokonta	Ascomyocota	*Umbilicaria pustulata (GCA_900169345)*	0	Genome	Ensembl Fungi
Opisthokonta	Basidiomycota	*Agaricus bisporus var. burnettii JB137-S8 (GCA_000300555)*	0	Genome	Ensembl Fungi
Opisthokonta	Basidiomycota	*Moniliophthora perniciosa FA553 (GCA_000183025)*	0	Genome	Ensembl Fungi
Opisthokonta	Basidiomycota	*Piloderma croceum F 1598 (GCA_000827315)*	0	Genome	Ensembl Fungi
Opisthokonta	Basidiomycota	*Coniophora puteana RWD-64-598 SS2 (GCA_000271625)*	0	Genome	Ensembl Fungi
Opisthokonta	Basidiomycota	*Suillus luteus UH-Slu-Lm8-n1 (GCA_000827255)*	0	Genome	Ensembl Fungi
Opisthokonta	Basidiomycota	*Botryobasidium botryosum FD-172 SS1 (GCA_000697705)*	0	Genome	Ensembl Fungi
Opisthokonta	Basidiomycota	*Rhizoctonia solani (GCA_001286725)*	0	Genome	Ensembl Fungi
Opisthokonta	Basidiomycota	*Punctularia strigosozonata HHB-11173 SS5 (GCA_000264995)*	0	Genome	Ensembl Fungi
Opisthokonta	Basidiomycota	*Xanthophyllomyces dendrorhous (GCA_001007165)*	0	Genome	Ensembl Fungi
Opisthokonta	Basidiomycota	*Calocera cornea HHB12733 (GCA_001632435)*	0	Genome	Ensembl Fungi
Opisthokonta	Basidiomycota	*Neolentinus lepideus HHB14362 ss-1 (GCA_001632425)*	0	Genome	Ensembl Fungi
Opisthokonta	Basidiomycota	*Fomitiporia mediterranea MF3/22 (GCA_000271605)*	0	Genome	Ensembl Fungi
Opisthokonta	Basidiomycota	*Jaapia argillacea MUCL 33604 (GCA_000697665)*	0	Genome	Ensembl Fungi
Opisthokonta	Basidiomycota	*Malassezia pachydermatis (GCA_001278385)*	0	Genome	Ensembl Fungi
Opisthokonta	Basidiomycota	*Mixia osmundae IAM 14324 (GCA_000241205)*	0	Genome	Ensembl Fungi
Opisthokonta	Basidiomycota	*Daedalea quercina L-15889 (GCA_001632345)*	0	Genome	Ensembl Fungi
Opisthokonta	Basidiomycota	*Melampsora larici-populina*	0	Genome	Ensembl Fungi
Opisthokonta	Basidiomycota	*Heterobasidion irregulare TC 32-1 (GCA_000320585)*	0	Genome	Ensembl Fungi
Opisthokonta	Basidiomycota	*Rhodotorula graminis WP1 (GCA_001329695)*	0	Genome	Ensembl Fungi
Opisthokonta	Basidiomycota	*Cryptococcus amylolentus CBS 6039 (GCA_001720205)*	0	Genome	Ensembl Fungi
Opisthokonta	Basidiomycota	*Kockovaella imperatae str. NRRL Y-17943 (GCA_002102565)*	0	Genome	Ensembl Fungi
Opisthokonta	Basidiomycota	*Anthracocystis flocculosa PF-1 (GCA_000417875)*	0	Genome	Ensembl Fungi
Opisthokonta	Basidiomycota	*Wallemia mellicola CBS 633.66 (GCA_000263375)*	0	Genome	Ensembl Fungi
Opisthokonta	Basidiobolomycota	*Basidiobolus meristosporus CBS 931.73 (GCA_002104905)*	0	Genome	Ensembl Fungi
Opisthokonta	Blastocladiomycota	*Allomyces macrogynus ATCC 38327 (GCA_000151295)*	0	Genome	Ensembl Fungi
Opisthokonta	Blastocladiomycota	*Catenaria anguillulae PL171 (GCA_002102555)*	0	Genome	Ensembl Fungi
Opisthokonta	Chytridiomyceta	*Anaeromyces robustus str. S4 (GCA_002104895)*	0	Genome	Ensembl Fungi
Opisthokonta	Chytridiomyceta	*Gonapodya prolifera JEL478 (GCA_001574975)*	0	Genome	Ensembl Fungi
Opisthokonta	Chytridiomyceta	*Neocallimastix californiae str. G1 (GCA_002104975)*	0	Genome	Ensembl Fungi
Opisthokonta	Chytridiomyceta	*Piromyces finnis (GCA_002104945)*	0	Genome	Ensembl Fungi
Opisthokonta	Chytridiomycota	*Rhizoclosmatium globosum str. JEL800 (GCA_002104985)*	0	Genome	Ensembl Fungi
Opisthokonta	Chytridiomycota	*Spizellomyces punctatus DAOM BR117 (GCA_000182565)*	0	Genome	Ensembl Fungi
Opisthokonta	Chytridiomycota	*Batrachochytrium dendrobatidis JAM81 (GCA_000203795)*	0	Genome	Ensembl Fungi
Opisthokonta	Chytridiomycota	*Batrachochytrium salamandrivorans str. BS (GCA_002006685)*	0	Genome	Ensembl Fungi
Opisthokonta	Cryptomycota	*Paramicrosporidium saccamoebae str. KSL3 (GCA_002794465)*	0	Genome	Ensembl Fungi
Opisthokonta	Cryptomycota	*Rozella allomycis CSF55 (GCA_000442015)*	0	Genome	Ensembl Fungi

Opisthokonta	Fonticulida	*Fonticula alba*	0	Genome	Ensembl Protists
Opisthokonta	Glomeromycota	*Rhizophagus clarus str. HR1*	0	Genome	Ensembl Fungi
Opisthokonta	Glomeromycota	*Rhizophagus irregularis DAOM 181602=DAOM 197198*	0	Genome	Ensembl Fungi
Opisthokonta	Microsporidia	*Amphiamblys sp. WSBS2006 (GCA_001875675)*	0	Genome	Ensembl Fungi
Opisthokonta	Microsporidia	*Edhazardia aedis USNM 41457 (GCA_000230595)*	0	Genome	Ensembl Fungi
Opisthokonta	Microsporidia	*Enterocytozoon bieneusi H348 (GCA_000209485)*	0	Genome	Ensembl Fungi
Opisthokonta	Microsporidia	*Enterocytozoon hepatopenaei str. TH1 (GCA_002081675)*	0	Genome	Ensembl Fungi
Opisthokonta	Microsporidia	*Enterospora canceri str. GB1 (GCA_002087915)*	0	Genome	Ensembl Fungi
Opisthokonta	Microsporidia	*Hepatospora eriocheir str. canceri (GCA_002087875)*	0	Genome	Ensembl Fungi
Opisthokonta	Microsporidia	*Hepatospora eriocheir str. GB1 (GCA_002087885)*	0	Genome	Ensembl Fungi
Opisthokonta	Microsporidia	*Mitosporidium daphniae str. UGP3 (GCA_000760515)*	0	Genome	Ensembl Fungi
Opisthokonta	Microsporidia	*Nematocida displodere str. JUm2807 (GCA_001642395)*	0	Genome	Ensembl Fungi
Opisthokonta	Microsporidia	*Nematocida parisii ERTm1 (GCA_000250985)*	0	Genome	Ensembl Fungi
Opisthokonta	Microsporidia	*Nematocida sp. ERTm5 (GCA_001642415)*	0	Genome	Ensembl Fungi
Opisthokonta	Microsporidia	*Pseudoloma neurophilia str. MK1 (GCA_001432165)*	0	Genome	Ensembl Fungi
Opisthokonta	Microsporidia	*Nosema apis BRL 01 (GCA_000447185)*	0	Genome	Ensembl Fungi
Opisthokonta	Microsporidia	*Nosema bombycis CQ1 (GCA_000383075)*	0	Genome	Ensembl Fungi
Opisthokonta	Microsporidia	*Nosema ceranae (GCA_000988165)*	0	Genome	Ensembl Fungi
Opisthokonta	Microsporidia	*Vittaforma corneae ATCC 50505 (GCA_000231115)*	0	Genome	Ensembl Fungi
Opisthokonta	Microsporidia	*Ordospora colligata OC4 (GCA_000803265)*	0	Genome	Ensembl Fungi
Opisthokonta	Microsporidia	*Trachipleistophora hominis (GCA_000316135)*	0	Genome	Ensembl Fungi
Opisthokonta	Microsporidia	*Vavraia culicis subsp. floridensis (GCA_000192795)*	0	Genome	Ensembl Fungi
Opisthokonta	Microsporidia	*Spraguea lophii 42_110 (GCA_000430065)*	0	Genome	Ensembl Fungi
Opisthokonta	Microsporidia	*Anncaliia algerae PRA109 (GCA_000385855)*	0	Genome	Ensembl Fungi
Opisthokonta	Microsporidia	*Encephalitozoon cuniculi EcunIII-L (GCA_001078035)*	0	Genome	Ensembl Fungi
Opisthokonta	Microsporidia	*Encephalitozoon hellem ATCC 50504 (GCA_000277815)*	0	Genome	Ensembl Fungi
Opisthokonta	Microsporidia	*Encephalitozoon intestinalis ATCC 50506 (GCA_000146465)*	0	Genome	Ensembl Fungi
Opisthokonta	Microsporidia	*Encephalitozoon romaleae SJ-2008 (GCA_000280035)*	0	Genome	Ensembl Fungi
Opisthokonta	Zoopagomyceta	*Conidiobolus coronatus NRRL 28638 (GCA_001566745)*	0	Genome	Ensembl Fungi
Opisthokonta	Zygomycota	*Lobosporangium transversale str. NRRL 3116*	0	Genome	Ensembl Fungi
Opisthokonta	Zygomycota	*Mortierella elongata AG-77*	0	Genome	Ensembl Fungi
Opisthokonta	Zygomycota	*Mortierella verticillata NRRL 6337 (GCA_000739165)*	0	Genome	Ensembl Fungi
Opisthokonta	Zygomycota	*Absidia repens str. NRRL 1336*	0	Genome	Ensembl Fungi
Opisthokonta	Zygomycota	*Bifiguratus adelaidae str. AZ0501*	0	Genome	Ensembl Fungi
Opisthokonta	Zygomycota	*Choanephora cucurbitarum str. KUS-F28377 (GCA_001683725)*	0	Genome	Ensembl Fungi
Opisthokonta	Zygomycota	*Hesseltinella vesiculosa str. NRRL 3301*	0	Genome	Ensembl Fungi
Opisthokonta	Zygomycota	*Lichtheimia corymbifera JMRC:FSU:9682 (GCA_000723665)*	0	Genome	Ensembl Fungi
Opisthokonta	Zygomycota	*Lichtheimia ramosa str. JMRC:FSU:6197 (GCA_000945115)*	0	Genome	Ensembl Fungi
Opisthokonta	Zygomycota	*Mucor ambiguus str. NBRC 6742 (GCA_000950595)*	0	Genome	Ensembl Fungi
Opisthokonta	Zygomycota	*Mucor circinelloides f. circinelloides 1006PhL (GCA_000401635)*	0	Genome	Ensembl Fungi
Opisthokonta	Zygomycota	*Rhizopus delemar RA 99-880 (GCA_000149305)*	0	Genome	Ensembl Fungi
Opisthokonta	Zygomycota	*Rhizopus microsporus str. CBS-344.29 (GCA_000825725)*	0	Genome	Ensembl Fungi
Opisthokonta	Zygomycota	*Syncephalastrum racemosum str. NRRL 2496*	0	Genome	Ensembl Fungi
Opisthokonta	Terestosporea	*Corallochytrium limacisporum*	0	Transcriptome	Torruella et al. 2015
Opisthokonta	Ichthyosporea	*Aboeforma whisleri*	0	Transcriptome	Torruella et al. 2015
Opisthokonta	Ichthyosporea	*Creolimax fragrantissima*	0	Transcriptome	Torruella et al. 2015
Opisthokonta	Ichthyosporea	*Ichthyophionus hoferi*	0	Transcriptome	Torruella et al. 2015
Opisthokonta	Ichthyosporea	*Pirum gemmata*	0	Transcriptome	Torruella et al. 2015
Opisthokonta	Ichthyosporea	*Sphaeroforma arctica*	0	Genome	Ensembl Protists
Opisthokonta	Ichthyosporea	*Sphaerothecum destruens*	0	Transcriptome	Torruella et al. 2015
Opisthokonta	Filasterea	*Capsaspora owczarzaki*	0	Genome	Ensembl Protists
Opisthokonta	Choanoflagellata	*Didymoeca costata*	3*	Transcriptome	Richter et al. 2018
Opisthokonta	Choanoflagellata	*Diaphanoeca grandis*	2*	Transcriptome	Richter et al. 2018
Opisthokonta	Choanoflagellata	*Monosiga brevicolis MX1*	0	Genome	Ensembl Protists
Opisthokonta	Choanoflagellata	*Salpingoeca infusionum*	3*	Transcriptome	Richter et al. 2018
Opisthokonta	Choanoflagellata	*Salpingoeca kvevrii*	6*	Transcriptome	Richter et al. 2018
Opisthokonta	Choanoflagellata	*Salpingoeca rosetta*	0	Genome	Ensembl Protists
Opisthokonta	Choanoflagellata	*Salpingoeca urceolata*	3*	Transcriptome	Richter et al. 2018

Table S3. Search for *Cthrc1* genes in available metazoan genomes and transcriptomes.

Clade	Species	# Cthrc1	Blasted data	Source
Ctenophora	*Pleurobrachia pileus*	0	Genome	Moroz et al. 2018
Ctenophora	*Mnemiopsis leidyi*	0	Genome	Ensembl Metazoa
Ctenophora	*Other transcriptomes*	0	Transcriptome	neurobase.rc.ufl.edu
Demospongiae	*Amphimedon queenslandica*	0	Genome	Ensembl Metazoa
Demospongiae	*Ephydatia fluviatiliis*	at least 1	Transcriptome	compagen.org
Demospongiae	*Stylissa carterri*	at least 2	Transcriptome	compagen.org
Homoscleromorpha	*Oscarella carmela*	4	Genome	compagen.org
Calcarea	*Sycon ciliatum*	at least 2	Transcriptome	compagen.org
Calcarea	*Leucosolenia complicata*	at least 2	Transcriptome	compagen.org
Placozoa	*Trichoplax adhaerens*	0	Genome	Ensembl Metazoa
Placozoa	*Hoilungia hongkongensis*	0	Genome	Eitel et al. 2018
Anthozoa	*Nematostella vectensis*	8	Genome	Ensembl Metazoa
Anthozoa	*Edwarsiella lineata*	6	Transcriptome	EdwardsiellaBase.org
Anthozoa	*Acropora digitifera*	18	Genome	NCBI nr
Anthozoa	*Orbicella faveolata*	31	Genome	NCBI nr
Anthozoa	*Stylophora pistillata*	19	Genome	NCBI nr
Hydrozoa	*Hydra vulgaris*	0	Genome	research.nhgri.nih.gov/hydra
Hydrozoa	*Clytia hemisphaerica*	14	Genome	Leclère et al. 2019
Scyphozoa	*Pelagia noctiluca*	at least 6	Transcriptome	Simion et al. 2017
Scyphozoa	*Aurelia spp.*	0	Genome	Gold et al. 2019<br>Khalturin et al. 2019
Cubozoa	*Morbakka virulenta*	0	Genome	Khalturin et al. 2019
Nemertodermatida	*Nemertoderma westbladi*	at least 1	Transcriptome	NCBI TSA
Xenoturbellida	*Xenoturbella bocki*	at least 1	Transcriptome	NCBI TSA
Acoela	*Hofstenia miamia*	0	Genome	Gehrke et al. 2019
Acoela	*Praesagittifera naikaiensis*	0	Genome	marinegenomics.oist.jp
Priapulida	*Priapulus caudatus*	at least 9	Transcriptome	NCBI nr
Nematoda	taxid: 6231	0	nr/nt/TSA	NCBI - 01/12/2019
Nematoda	*Caenorhabditis brenneri*	0	Genome	Ensembl Metazoa
Nematoda	*Caenorhabditis briggsae*	0	Genome	Ensembl Metazoa
Nematoda	*Caenorhabditis elegans*	0	Genome	Ensembl
Nematoda	*Caenorhabditis japonica*	0	Genome	Ensembl Metazoa
Nematoda	*Caenorhabditis remanei*	0	Genome	Ensembl Metazoa
Nematoda	*Brugia malayi*	0	Genome	Ensembl Metazoa
Nematoda	*Loa loa*	0	Genome	Ensembl Metazoa
Nematoda	*Onchocerca volvulus*	0	Genome	Ensembl Metazoa
Nematoda	*Pristionchus pacificus*	0	Genome	Ensembl Metazoa
Nematoda	*Strongyloides ratti*	0	Genome	Ensembl Metazoa
Arthropoda	*Sarcoptes scabiei*	0	Genome	Ensembl Metazoa
Arthropoda	*Stegodyphus mimosarum*	0	Genome	Ensembl Metazoa
Arthropoda	*Ixodes scapularis*	0	Genome	Ensembl Metazoa
Arthropoda	*Tetranychus urticae*	0	Genome	Ensembl Metazoa
Arthropoda	taxid: 6656	0	nr/nt/TSA	NCBI - 01/12/2019
Onychophora	taxid: 27563	0	nr/nt/TSA	NCBI - 01/12/2019
Tardigrade	*Ramazzottius varieornatus*	0	Genome	kumamushi.org
Platyhelminthes	*Schmidtea mediterranea*	0	Genome	smedgd.stowers.org
Platyhelminthes	*Macrostomum lignano*	0	Genome	macgenome.org
Platyhelminthes	*Echinococcus multilocularis*	0	Genome	sanger.ac.uk
Brachiopoda	*Lingula anatina*	8**	Genome	NCBI nr/ TSA

Brachiopoda	*Novocrania anomala*	at least 1	EST	NCBI EST
Bryozoa	*Tubulipora sp.*	at least 1	EST	NCBI EST
Phoronida	*Phoronida australis*	at least 3**	Transcriptome	NCBI TSA
Nemertean	*Notospermus geniculatus*	0	Genome	marinegenomics.oist.jp
Annelida	*Platynereis dumerilii*	1	Genome	Private website
Annelida	*Lamellibrachia satsuma*	at least 1	Transcriptome	NCBI TSA
Annelida	*Pygospia elegans*	at least 1	Transcriptome	NCBI TSA
Annelida	*Perinereis aibuhhitensis*	at least 1	Transcriptome	NCBI TSA
Annelida	*Capitella teleta*	0	Genome	Ensembl Metazoa
Annelida	*Helobdella robusta*	0	Genome	Ensembl Metazoa
Rotifera	*Adineta vaga*	0	Genome	Ensembl Metazoa
Mollusca	*Crassostrea gigas*	0	Genome	Ensembl Metazoa
Mollusca	*Lottia gigantea*	0	Genome	Ensembl Metazoa
Mollusca	*Octopus bimaculoides*	0	Genome	Ensembl Metazoa
Mollusca	*Pinctada fucata*	0	Genome	marinegenomics.oist.jp
Mollusca	taxid:6447	0	nr/nt/TSA	NCBI - 01/12/2019
Echinodermata	*Strongylocentrotus purpuratus*	6	Genome	NCBI nr/echinobase.org
Echinodermata	*Paracentrotus lividus*	at least 2	Transcriptome	NCBI nr
Echinodermata	*Apostichopus japonicus*	3	Genome	NCBI nr
Hemichordata	*Saccoglossus kowalevskii*	at least 2	Genome	NCBI nr
Hemichordata	*Ptychodera flava*	6***	Genome	marinegenomics.oist.jp
Cephalochordata	*Branchiostoma floridae*	18	Genome	JGI MycoCosm
Urochordata	*Ciona intestinalis*	1	Genome	NCBI nr/Aniseed
Urochordata	*Phallusia mammilata*	1	Genome	NCBI nr/Aniseed
Urochordata	*Botryllus schlosseri*	0	Genome	Stanford database
Urochordata	*Botryloides leichii*	0	Genome	Alié et al. 2018
Urochordata	*Distomus variolosus*	at least 1	Transcriptome	Alié et al. 2018
Urochordata	*Polyandrocarpa zorritensis*	at least 1	Transcriptome	Alié et al. 2018
Urochordata	*Asterocarpa humilis*	at least 1	Transcriptome	Alié et al. 2018
Urochordata	*Oikopleura dioica*	0	Genome	OikoBase
Petromyzontiformes	*Petromyzon marinus*	1 - partial	Genome	Ensembl
Gnathostomes	taxid: 7776	1 to 4	nr/nt	NCBI - 01/12/2019

Notes for Table S2 and S3:

*: None of the predicted proteins include a CTHR domain.
**: Others predicted proteins including the Cthrc1 C-terminal domain without the CTHR domain.
***: Other predicted proteins seem partial and were not counted.
Ensembl Protists: release 45 http://protists.ensembl.org/index.html
Ensembl Fungi: release 45 http://fungi.ensembl.org/index.html
Ensembl Plants: release 45 https://plants.ensembl.org/index.html
Ensembl Metazoa: release 45 https://metazoa.ensembl.org
Ensembl: release 98 https://www.ensembl.org/
PlantGDB: http://www.plantgdb.org/
Stanford database: http://botryllus.stanford.edu/botryllusgenome/
Aniseed: https://www.aniseed.cnrs.fr/
OikoBase: http://oikoarrays.biology.uiowa.edu/
JGI MyoCosm https://mycocosm.jgi.doe.gov/
EdwardBase: http://cnidarians.bu.edu/EdwardBase/cgi-bin/index.cgi

Supplementary references:

Eitel et al. 2018. Comparative genomics and the nature of placozoan species. PLoS Biol. 16(7):e2005359.

Moroz et al. 2014. The ctenophore genome and the evolutionary origins of neural systems. Nature. 510(7503):109-14.

Stephens et al. 2019. *Polarella glacialis* genomes encode tandem repeats of single-exon genes with functions critical to adaptation of dinoflagellates. bioRxiv 704437.

Table S4. *Cthrc1* X2 isoform in primates as found in the NCBI databases.

Species name	Common Name	Group	X2?	NCBI accession	X2 exon sequence
Homo sapiens	Man	Hominidae	Yes	NP_001243028.1	MWPPGRSITVKLREKTVSRKLEMNGPSAFQGLICGK
Pan troglodytes	Chimpanzee	Hominidae	Yes	XP_003951375	MWPPGRSITVKLREKTVSRKLEMNGLSAFQGLICGK
Gorilla gorilla	Gorilla	Hominidae	Yes	XP_004047456	MWPPGRSITVKLREKTVSRKLEMNGPSAFQGLICGK
Pongo abelii	Orangutan	Hominidae	Yes	XP_003777340	MWPPGRSITVKLGEKTVSRKLEMNGPSAFQGLICGK
Nomascus leucogenys	Gibbon	Hylobatidae	Yes	None	MWPPGRSITVKLWEKTVSRKLEMNGPSAFQGLVCGN*
Mandrillus leucophaeus	Drill	Old World Monkeys	Yes	XP_011838895	MWTPGRSITVKLGEKTVSRKLEMNGRGAFQGLICGK
Macaca fascicularis	Crab-eating macaque	Old World Monkeys	Yes	XP_005563912	MWPPGRSITVKLGEKTVSRKLEMNGRGAFQGLICGK
Macaca mulatta	Rhesus macaque	Old World Monkeys	Yes	XP_015001294.2	MWPPGRSITVKLGEKTVSRKLEMNGRGAFQGLICGK
Piliocolobus tephrosceles	Red Colobus monkey	Old World Monkeys	Yes	XP_023081197	MWPPGRSITVKLGEKTVSRKLEMNGRSAFQGLICGK
Rhinopithecus bieti	Black snub-nosed monkey	Old World Monkeys	Yes	None	MWPPGRSITVKLGEKTVSRKLEMNGRSAFQGLICGK
Rhinopithecus roxellana	Golden snub-nosed monkey	Old World Monkeys	Yes	None	MWPPGRSITVKLGEKTVSRKLEMNGRSAFQGLICGK
Colobus angolensis palliates	Angola Colobus	Old World Monkeys	Yes	None	MWPPGRSITVKLGEKTVSRKLEMNGRSAFQGLICGK
Cercocebus atys	Mangabey	Old World Monkeys	Yes	XP_011917110	MNGRGAFQGLICGK
Papio anubis	Olive baboon	Old World Monkeys	Yes	None	MNGRGAFQGLICGK
Theropithecus gelada	Gelada "Baboon"	Old World Monkeys	Yes	None	MNGRGAFQGLICGK
Callithrix jacchus	Marmoset	New World Monkeys	No		X2 like sequence NOT found
Cebus capucinus	Capuchin	New World Monkeys	No		X2 like sequence NOT found
Saimiri boliviensis	Squirrel monkey	New World Monkeys	No		X2 like sequence NOT found
Aotus nancymaae	Night monkey	New World Monkeys	No		X2 like sequence NOT found
Propithecus coquereli	Sifaka	Lemurs	No		X2 like sequence NOT found
Otolemur garnettii	Small eared galago (Bushbaby)	Galagos	No		X2 like sequence NOT found
Nycticebus coucang	Slow loris	Lorises	No		X2 like sequence NOT found

Notes for Table S4:

*: No intron donor sequence.

In red: Amino-acid differences as compared to human.
